# Prediction of resistance to chemotherapy in ovarian cancer: a systematic review

**DOI:** 10.1186/s12885-015-1101-8

**Published:** 2015-03-11

**Authors:** Katherine L Lloyd, Ian A Cree, Richard S Savage

**Affiliations:** 1 MOAC DTC, University of Warwick, Gibbet Hill Road, Coventry, CV4 7AL UK; 2 Warwick Medical School, University of Warwick, Gibbet Hill Road, Coventry, CV4 7AL UK; 3 Systems Biology Centre, University of Warwick, Gibbet Hill Road, Coventry, CV4 7AL UK

**Keywords:** Ovarian cancer, Chemoresistance, Predictive model, Statistical modelling

## Abstract

**Background:**

Patient response to chemotherapy for ovarian cancer is extremely heterogeneous and there are currently no tools to aid the prediction of sensitivity or resistance to chemotherapy and allow treatment stratification. Such a tool could greatly improve patient survival by identifying the most appropriate treatment on a patient-specific basis.

**Methods:**

PubMed was searched for studies predicting response or resistance to chemotherapy using gene expression measurements of human tissue in ovarian cancer.

**Results:**

42 studies were identified and both the data collection and modelling methods were compared. The majority of studies utilised fresh-frozen or formalin-fixed paraffin-embedded tissue. Modelling techniques varied, the most popular being Cox proportional hazards regression and hierarchical clustering which were used by 17 and 11 studies respectively. The gene signatures identified by the various studies were not consistent, with very few genes being identified by more than two studies. Patient cohorts were often noted to be heterogeneous with respect to chemotherapy treatment undergone by patients.

**Conclusions:**

A clinically applicable gene signature capable of predicting patient response to chemotherapy has not yet been identified. Research into a predictive, as opposed to prognostic, model could be highly beneficial and aid the identification of the most suitable treatment for patients.

**Electronic supplementary material:**

The online version of this article (doi:10.1186/s12885-015-1101-8) contains supplementary material, which is available to authorized users.

## Background

Ovarian cancer is the fifth most common cancer in women in the UK and accounted for 4% of cancer diagnoses in women between 2008 and 2010 [1]. Worryingly, it was also responsible for 6% of cancer-related deaths in women over the same time period [1] and the five-year survival of women diagnosed with ovarian cancer between 2005 and 2009 was 42% [2]. It has been observed that although 40%-60% of patients achieve complete clinical response to first-line chemotherapy treatment [3], around 50% of these patients relapse within 5 years [4] and only 10%-15% of patients presenting with advanced stage disease achieve long-term remission [5]. It is thought that the high relapse rate is at least in part due to resistance to chemotherapy, which may be inherent or acquired by altered gene expression [6].

For ovarian cancer in the UK, the standard of care for first-line chemotherapy treatment recommended by the National Institute for Health and Care Excellence is ‘paclitaxel in combination with a platinum-based compound or platinum-based therapy alone’ [7]. This uniform approach ignores the complexity of ovarian cancer histologic types, particularly as there is evidence to suggest differences in response [8]. Winter *et al.* [9] investigated the survival of patients following paclitaxel and platinum chemotherapy and found histology to be a significant predictor of overall survival in multivariate Cox proportional hazards regression.

Improvement in survival has also been poor in ovarian cancer. Between 1971 and 2007 there was a 38% increase in relative 10-year survival in breast cancer, whereas the increase in ovarian cancer was 17% [10]. This difference in progress is likely to be due, at least in part, to the lack of tools with which to predict chemotherapy response in ovarian cancer.

Gene expression based tools for the prediction of patient prognosis after surgery or chemotherapy are currently available for some cancers. For example, MammaPrint^®;^ uses the expression of 70 genes to predict the likelihood of metastasis in breast cancer [11]. Similarly, the Oncotype DX^®;^ assay uses the expression of a panel of 21 genes to predict recurrence after treatment of breast cancer [12]. The Oncotype DX assay is also available for colon [13] and prostate cancers [14]. The development of a similar tool for ovarian cancer could greatly improve patient prognosis and quality of life by guiding chemotherapy choices. The prediction of cancer prognosis using gene signatures is a popular research field, within which a wide variety of approaches have been considered. Popular RNA or protein expression measurement techniques include cDNA hybridisation microarrays, end-point and quantitative reverse transcription PCR, and immunohistochemistry approaches.

Another variable aspect of studies predicting chemotherapy response is the computational and statistical approaches utilised. One of most popular methods for survival analysis is Cox proportional hazards regression. This model assumes that the hazard of death is proportional to the exponential of a linear predictor formed of the explanatory variables. This model has the advantage that, unlike many other regression techniques, it can appropriately deal with right-censored data such as that found in medical studies where patients leave before the end of the study period [15].

Other popular modelling techniques include linear models, support vector machines, hierarchical clustering, principal components analysis and the formation of a scoring algorithm. When dealing with data sets of varying sizes it is important to consider the number of samples and the amount of data per patient when choosing a modelling method. If the number of patients is large it is clear that a model will be better informed about the population from which the patient sample was drawn, and hence is likely to generalise more effectively to independent data sets. As the number of measurements per patient increases, the dimensionality and hence the flexibility of the model may increase. However, it is also important that the number of patients is sufficiently large to supply enough information about the factors being considered. Of the models identified here, linear models are relatively restrictive as the relationship between any factor and the outcome is assumed to be linear and so are suitable for smaller data sets. Conversely, hierarchical clustering simply finds groups of similar samples and there are minimal assumptions concerning the relationship between factors and outcome.

Classification models are used to predict which of a number of groups an individual falls into and are used for categorical variables, such as tumour grade and having or not having a disease. For visualisation and the assessment of classification model predictive power, a Kaplan-Meier plot is often combined with the log-rank test to investigate significance. It is worth noting that this method does not compare predictions with measurements, it simply considers the difference in survival between groups.

Many of the studies identified by this review involved developing a model using one set of samples, a training set, followed by testing of the model carried out on an independent set of samples, the test or validation set. This partitioning of samples is important as it allows the generalisability of the model to be assessed, and hence guards against over-fitting. If this check is not carried out, the true predictive ability of the model will not be known.

The aim of this review is to investigate the literature surrounding the prediction of chemotherapy response in ovarian cancer using gene expression. It has been observed, for example by Gillet *et al*. [16], that gene signatures obtained from cancer cell lines are not always relevant to *in vivo* studies, and that cell lines are inaccurate models of chemosensitivity [17]. The search was therefore restricted to studies involving human tissue in order to ensure that the resulting gene signatures are applicable in a clinical setting. It was also specified that the study must involve patients who have undergone chemotherapy treatment, so that the effects of resistance may be investigated.

## Methods

### Search methodology

The aim of this review is to investigate the literature on the prediction of chemoresistance in patients with ovarian cancer. Therefore, the six most important requirements identified were:

- Concerned with (specifically) ovarian cancer
- Patients were treated with chemotherapy
- Gene expression was measured for use in predictions
- Predictions are related to a measure of chemoresistance (e.g. response rates, progression-free survival)
- Measurements were taken on human tissue (not cell lines)
- The research aim is to develop a diagnostic tool or predict response

A PubMed search was carried out on 6th August 2014 to identify studies fulfilling the above requirements. The search terms may be found in Additional file 1. This search resulted in 78 papers.

### Filtering

**Figure 1:**
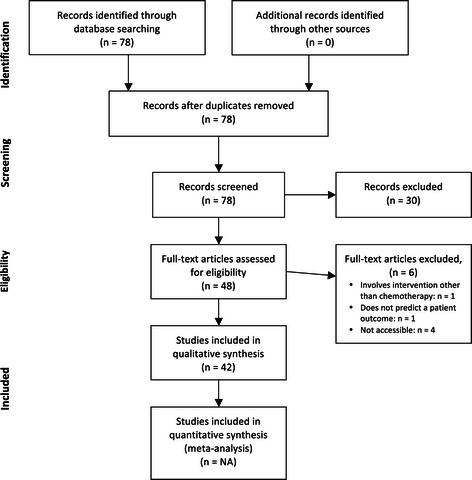
**PRISMA search filtering flow diagram.** The initial search results were filtered using titles and abstracts and, later, the full text to ensure the search criteria were fulfilled. Following filtering the number of papers included reduced from 78 to 42.

The search results were filtered twice, once based on abstracts and once based on full texts, by KL. An overview of the filtering process may be found in Figure 1. For the abstract-based filtering, papers were excluded if the six essential criteria were not all met, if the paper was a review article or if the paper was non-English language. This resulted in 48 papers remaining. For the full-text-based filtering, exclusion was due to not fulfilling the search criteria or papers that were not available. 42 papers were remaining after full-text-based filtering.

### Data extraction

Data was extracted using a pre-defined table created for the purpose. Extraction was carried out in duplicate by a single author (KL) with a wash-out period of 3 months to avoid bias. Variables extracted were: author, year, journal, number of samples, number of genes measured, study end-point, tissue source, percentage cancerous tissue, gene or protein expression measurement technique, sample histological types and stages, patient prior chemotherapy, modelling techniques applied, whether the model accounts for heterogeneity in patient chemotherapy, whether the model was prognostic or predictive, whether the model was validated, model predictive ability including any metrics or statistics, and the genes found to be predictive.

### Bias analysis

Bias in the studies selected for the systematic review was assessed according to QUADAS-2 [18], a tool for the quality assessment of diagnostic accuracy studies. Levels of evidence were also assessed according to the CEBM 2011 Levels of Evidence [19]. Results of these analyses may be found in Additional files 2 and 3. Briefly, the majority of studies were considered to be low risk, with six studies judged to have unclear risk for at least one domain and seven studies judged to be high risk for at least one domain. Thirty-six studies where judged to have evidence of level 2, with the remaining six having evidence of level 3. These levels of risk and evidence suggest that the majority of conclusions drawn from these studies are representative and applicable to the review question.

### Gene set enrichment

Gene set enrichment analysis was applied to the gene sets reported by the studies selected for this review. Analysis was performed using the R package *HTSanalyseR* [20]. Where reported, gene sets were extracted and combined according to the chemotherapy treatments applied to patients in each study. The two groups assessed were those studies where all patients were treated with platinum and taxane in combination, and those studies where patients were given treatments other than platinum and taxane. The second group includes those given platinum as a single agent. Any studies reporting treatments from both groups were excluded, as were studies that did not report the chemotherapy treatments used. Kyoto Encyclopedia of Genes and Genomes (KEGG) terms were identified for each gene and gene set collection analysis was carried out, which applies hypergeometric tests and gene set enrichment analysis. A p-value cut-off of 0.0001 was used. Enrichment maps were then plotted, using the 30 most significant KEGG terms. P-values were adjusted using the ‘BH’ correction [21].

### Ethics statement

Ethical approval was not required for this systematic review, which deals exclusively with previously published data.

## Results

Tables 1, 2, 3, 4, 5 and 6 detail some key information regarding the studies included in the review. Table 1 contains the number of samples analysed, the number of genes considered for the model, and the resulting genes retained as the predictive gene signature. Table 2 provides information about the tissue used for gene expression measurements and whether the studies assessed the percent neoplastic tissue before measurement, and Table 3 details the gene expression measurement techniques used. Table 4 contains the reported histological types and stages of the samples processed by each study. Table 5 provides information on chemotherapy treatments undergone by patients, whether the model was prognostic or predictive, and whether the model was validated using either an independent set of samples or cross validation. Table 6 lists the outcome to be predicted, the modelling techniques applied, and the predictive ability of the resulting model.

**Table 1:** **Journal and study information of papers included in the systematic review**

Study	Journal	No. samples	No. genes in study	No. genes in signature
Jeong *et al.* [22]	Anticancer Res.	487	612	388, 612
Lisowska *et al.* [23]	Front. Oncol.	127	>47000	0
Roque *et al.* [24]	Clin. Exp. Metastasis	48	1	1
Li *et al.* [3]	Oncol. Rep.	44	1	1
Schwede *et al.* [25]	PLoS ONE	663	2632	51
Verhaak *et al.* [26]	J. Clin. Invest.	1368	11861	100
Obermayr *et al.* [27]	Gynecol. Oncol.	255	29098	12
Han *et al.* [28]	PLoS ONE	322	12042	349, 18
Hsu *et al.* [29]	BMC Genomics	168	12042	134
Lui *et al.* [30]	PLoS ONE	737	NS	227
Kang *et al.* [31]	J. Nat. Cancer Inst.	558	151	23
Gillet *et al.* [32]	Clin. Cancer Res.	80	356	11
Ferriss *et al.* [33]	PLos ONE	341	NS	251, 125
Brun *et al.* [34]	Oncol. Rep.	69	6	0
Skirnisdottir and Seidal [35]	Oncol. Rep.	105	3	2
Brenne *et al.* [36]	Hum. Pathol.	140	1	1
Sabatier *et al.* [37]	Br. J. Cancer	401	NS	7
Gillet *et al.* [38]	Mol. Pharmeceutics	32	350	18, 10, 6
Chao *et al.* [39]	BMC Med. Genomics	6	8173	NS
Schlumbrecht *et al.* [40]	Mod. Pathol.	83	7	2
Glaysher *et al.* [41]	Br. J. Cancer	31	91	10, 4, 3, 5, 5, 11, 6, 6
Yan *et al.* [42]	Cancer Res.	42	2	1
Yoshihara *et al.* [43]	PLoS ONE	197	18176	88
Williams *et al.* [44]	Cancer Res.	242	NS	15 to 95
Denkert *et al.* [45]	J. Pathol	198	NS	300
Matsumura *et al.* [46]	Mol. Cancer Res.	157	22215	250
Crijns *et al.* [47]	PLoS Medicine	275	15909	86
Mendiola *et al.* [48]	PLoS ONE	61	82	34
Gevaert *et al.* [49]	BMC Cancer	69	∼24000	∼3000
Bachvarov *et al.* [50]	Int. J. Oncol.	42	20174	155, 43
Netinatsunthorn *et al.* [51]	BMC Cancer	99	1	1
De Smet *et al.* [52]	Int. J. Gynecol. Cancer	20	21372	3000
Helleman *et al.* [53]	Int. J. Cancer	96	NS	9
Spentzos *et al.* [54]	J. Clin. Oncol.	60	NS	93
Jazaeri *et al.* [55]	Clin. Cancer Res.	40	40033, 7585	85, 178
Raspollini *et al.* [56]	Int. J. Gynecol. Cancer	52	2	2
Hartmann *et al.* [57]	Clin. Cancer Res.	79	30721	14
Spentzos *et al.* [58]	J. Clin. Oncol.	68	12625	115
Selvanayagam *et al.* [59]	Cancer Genet. Cytogenet.	8	10692	NS
Iba *et al.* [60]	Cancer Sci.	118	4	1
Kamazawa *et al.* [61]	Gynecol. Oncol.	27	3	1
Vogt *et al.* [62]	Acta Biochim. Pol.	17	3	0

If more than one value is given, the study used multiple different starting gene-sets or found multiple gene signatures. NS: Not Specified.

**Table 2:** **Tissue information of papers included in systematic review**

Study	Tissue source	% Cancerous tissue
Jeong *et al.* [22]		
Lisowska *et al.* [23]	Fresh-frozen	NS
Roque *et al.* [24]	FFPE, Fresh-frozen	min. 70%
Li *et al.* [3]	FFPE	NS
Schwede *et al.* [25]		
Verhaak *et al.* [26]		
Obermayr *et al.* [27]	Fresh-frozen, Blood	NS
Han *et al.* [28]		
Hsu *et al.* [29]		
Lui *et al.* [30]		
Kang *et al.* [31]		
Gillet *et al.* [32]	Fresh-frozen	min. 75%
Ferriss *et al.* [33]	FFPE	min. 70%
Brun *et al.* [34]	FFPE	NS
Skirnisdottir and Seidal [35]	FFPE	NS
Brenne *et al.* [36]	Fresh-frozen effusion, Fresh-frozen	min. 50%
Sabatier *et al.* [37]	Fresh-frozen	min. 60%
Gillet *et al.* [38]	Fresh-frozen effusion	NS
Chao *et al.* [39]		
Schlumbrecht *et al.* [40]	Fresh-frozen	min. 70%
Glaysher *et al.* [41]	FFPE, Fresh	min. 80%
Yan *et al.* [42]	Fresh-frozen	NS
Yoshihara *et al.* [43]	Fresh-frozen	min. 80%
Williams *et al.* [44]		
Denkert *et al.* [45]	Fresh-frozen	NS
Matsumura *et al.* [46]	Fresh-frozen	NS
Crijns *et al.* [47]	Fresh-frozen	median = 70%
Mendiola *et al.* [48]	FFPE	min. 80%
Gevaert *et al.* [49]	Fresh-frozen	NS
Bachvarov *et al.* [50]	Fresh-frozen	min. 70%
Netinatsunthorn *et al.* [51]	FFPE	NS
De Smet *et al.* [52]	Not specified	NS
Helleman *et al.* [53]	Fresh-frozen	median = 64%
Spentzos *et al.* [54]	Fresh-frozen	NS
Jazaeri *et al.* [55]	FFPE, Fresh-frozen	NS
Raspollini *et al.* [56]	FFPE	NS
Hartmann *et al.* [57]	Fresh-frozen	min. 70%
Spentzos *et al.* [58]	Fresh-frozen	NS
Selvanayagam *et al.* [59]	Fresh-frozen	min. 70%
Iba *et al.* [60]	FFPE, Fresh-frozen	NS
Kamazawa *et al.* [61]	FFPE, Fresh-frozen	NS
Vogt *et al.* [62]	None specified	NS

If more than one value is given, the study used tissue from multiple sources. NS: Not Specified.

**Table 3:** **Gene expression measurement techique information of papers included in systematic review**

Study	Immunohistochemistry	TaqMan array	q-RT-PCR	Commercial microarray	Custom microarray	RT-PCR
Jeong *et al.* [22]	✗	✗	✗	✓	✗	✗
Lisowska *et al.* [23]	✗	✗	✓	✓	✗	✗
Roque *et al.* [24]	✓	✗	✓	✗	✗	✗
Li *et al.* [3]	✓	✗	✗	✗	✗	✗
Schwede *et al.* [25]	✗	✗	✗	✓	✗	✗
Verhaak *et al.* [26]	✗	✗	✗	✓	✗	✗
Obermayr *et al.* [27]	✗	✗	✓	✓	✗	✗
Han *et al.* [28]	✗	✗	✗	✓	✗	✗
Hsu *et al.* [29]	✗	✗	✗	✓	✗	✗
Lui *et al.* [30]	✗	✗	✗	✓	✗	✗
Kang *et al.* [31]	✗	✗	✗	✓	✗	✗
Gillet *et al.* [32]	✗	✓	✗	✗	✗	✗
Ferriss *et al.* [33]	✗	✗	✗	✗	✓	✗
Brun *et al.* [34]	✓	✗	✗	✗	✗	✗
Skirnisdottir and Seidal [35]	✓	✗	✗	✗	✗	✗
Brenne *et al.* [36]	✗	✗	✓	✗	✗	✗
Sabatier *et al.* [37]	✗	✗	✗	✓	✗	✗
Gillet *et al.* [38]	✗	✓	✗	✗	✗	✗
Chao *et al.* [39]	✗	✗	✗	✓	✗	✗
Schlumbrecht *et al.* [40]	✓	✗	✓	✗	✗	✗
Glaysher *et al.* [41]	✗	✓	✗	✗	✗	✗
Yan *et al.* [42]	✓	✗	✗	✗	✗	✗
Yoshihara *et al.* [43]	✗	✗	✓	✓	✗	✗
Williams *et al.* [44]	✗	✗	✗	✓	✗	✗
Denkert *et al.* [45]	✗	✗	✗	✓	✗	✗
Matsumura *et al.* [46]	✓	✗	✓	✓	✗	✗
Crijns *et al.* [47]	✗	✗	✓	✗	✓	✗
Mendiola *et al.* [48]	✗	✓	✗	✗	✗	✗
Gevaert *et al.* [49]	✗	✗	✗	✓	✗	✗
Bachvarov *et al.* [50]	✗	✗	✓	✓	✗	✗
Netinatsunthorn *et al.* [51]	✓	✗	✗	✗	✗	✗
De Smet *et al.* [52]	✗	✗	✗	✗	✓	✗
Helleman *et al.* [53]	✗	✗	✓	✗	✓	✗
Spentzos *et al.* [54]	✗	✗	✗	✓	✗	✗
Jazaeri *et al.* [55]	✓	✗	✗	✗	✓	✗
Raspollini *et al.* [56]	✓	✗	✗	✗	✗	✗
Hartmann *et al.* [57]	✗	✗	✗	✗	✓	✗
Spentzos *et al.* [58]	✗	✗	✗	✓	✗	✗
Selvanayagam *et al.* [59]	✗	✗	✗	✗	✓	✗
Iba *et al.* [60]	✓	✗	✓	✗	✗	✗
Kamazawa *et al.* [61]	✗	✗	✓	✗	✗	✗
Vogt *et al.* [62]	✗	✗	✗	✗	✗	✓

**Table 4:** **Histology information of papers included in systematic review**

Study	Sub-type	Stage
Jeong *et al.* [22]	**Serous**, Endometrioid, Adenocarcinoma	I, II, III, IV
Lisowska *et al.* [23]	Serous, Endometrioid, Clear cell, Undifferentiated	II, **III**, IV
Roque *et al.* [24]	Serous, Endometrioid, Clear cell, Undifferentiated, Mixed	IIIC, IV
Li *et al.* [3]	Serous, Endometrioid, Clear cell, Mucinous, Transitional	II, III, IV
Schwede *et al.* [25]	Serous, Endometrioid, Clear cell, Mucinous, Adenocarcinoma, OSE	I, II, III, IV
Verhaak *et al.* [26]	NS	II, III, IV
Obermayr *et al.* [27]	**Serous**, Non-serous	II, III, IV
Han *et al.* [28]	**Serous**, Endometrioid, Clear cell, Mucinous, Mixed, Poorly differentiated	II, III, IV
Hsu *et al.* [29]	NS	**III**, IV
Lui *et al.* [30]	**Serous**	II, **III**, IV
Kang *et al.* [31]	**Serous**	I, II, III, IV
Gillet *et al.* [32]	**Serous**	**III**, IV
Ferriss *et al.* [33]	**Serous**, Clear cell, Other	**III**, IV
Brun *et al.* [34]	Serous, Endometrioid, Clear cell, Mucinous, Other	III, IV
Skirnisdottir and Seidal [35]	Serous, Endometrioid, Clear cell, Mucinous, Anaplastic	I, II
Brenne *et al.* [36]	**Serous**, Endometrioid, Clear cell, Undifferentiated, Mixed	II, III, IV
Sabatier *et al.* [37]	Serous, Endometrioid, Clear cell, Mucinous, Undifferentiated, Mixed	I, II, III, IV
Gillet *et al.* [38]	**Serous**	III, IV, NS
Chao *et al.* [39]	NS	NS
Schlumbrecht *et al.* [40]	**Serous**	III, IV
Glaysher *et al.* [41]	Serous, Endometrioid, Clear cell, Mucinous, Mixed, Poorly differentiated	IIIC, IV
Yan *et al.* [42]	Serous, Endometrioid, Clear cell, Mucinous, Transitional	II, **III**, IV
Yoshihara *et al.* [43]	**Serous**	**III**, IV
Williams *et al.* [44]	**Serous**, Endometrioid, Undifferentiated	**III**, IV
Denkert *et al.* [45]	**Serous**, Non-serous, Undifferentiated	I, II, **III**, IV
Matsumura *et al.* [46]	**Serous**	I, II, III, IV
Crijns *et al.* [47]	**Serous**	**III**, IV
Mendiola *et al.* [48]	Serous, Non-serous	**III**, IV
Gevaert *et al.* [49]	**Serous**, Endometrioid, Mucinous, Mixed	I, III, IV
Bachvarov *et al.* [50]	**Serous**, Endometrioid, Clear cell	II, III, IV
Netinatsunthorn *et al.* [51]	**Serous**	**III**, IV
De Smet *et al.* [52]	Serous, Endometrioid, Mucinous, Mixed	I, III, IV
Helleman *et al.* [53]	Serous, Endometrioid, Clear cell, Mucinous, Mixed, Poorly differentiated	I/II, **III/IV**
Spentzos *et al.* [54]	**Serous**, Endometrioid, Clear cell, Mixed	I, II, **III**, IV
Jazaeri *et al.* [55]	**Serous**, Endometrioid, Clear cell, Mixed, Undifferentiated, Carcinoma	II, III, IV
Raspollini *et al.* [56]	**Serous**	**IIIC**
Hartmann *et al.* [57]	Serous, Endometrioid, Mixed	II, III, IV
Spentzos *et al.* [58]	**Serous**, Endometrioid, Clear cell, Mixed	I, II, **III**, IV
Selvanayagam *et al.* [59]	Serous, Endometrioid, Clear cell, Undifferentiated	**III**, IV
Iba *et al.* [60]	Serous, Endometrioid, Clear cell, Mixed	I, II, III, IV
Kamazawa *et al.* [61]	**Serous**, Endometrioid, Clear cell	III, IV
Vogt *et al.* [62]	NS	NS

Entries in bold indicate that the study data set was comprised of at least 80% this type. NS: Not Specified.

**Table 5:** **Basic modelling and patient information of papers included in systematic review**

Study	Patient prior chemotherapy treatment	Model accounts for the different chemotherapies?	Prognostic or predictive?	Model validated?
Jeong *et al.* [22]	Platinum-based	✓	Predictive	✓
Lisowska *et al.* [23]	Platinum/Cyclophosphamide, Platinum/Taxane	✗	Prognostic	✓
Roque *et al.* [24]	NS	✗	Prognostic	✗
Li *et al.* [3]	Platinum/Cyclophosphamide, Platinum/Taxane	✗	Prognostic	✗
Schwede *et al.* [25]	NS	✗	Prognostic	✓
Verhaak *et al.* [26]	NS	✗	Prognostic	✓
Obermayr *et al.* [27]	Platinum-based	✗	Prognostic	✗
Han *et al.* [28]	Platinum/Paclitaxel		Prognostic	✓
Hsu *et al.* [29]	Platinum/Paclitaxel			
	+ additional treatments	✓	Prognostic	✓
Lui *et al.* [30]	NS	✗	Prognostic	✓
Kang *et al.* [31]	Platinum/Taxane		Prognostic	✓
Gillet *et al.* [32]	Carboplatin/Paclitaxel		Prognostic	✓
Ferriss *et al.* [33]	Platinum-based	✓	Predictive	✓
Brun *et al.* [34]	NS	✗	Prognostic	✗
Skirnisdottir and Seidal [35]	Carboplatin/Paclitaxel		Prognostic	✗
Brenne *et al.* [36]	NS	✗	Prognostic	✗
Sabatier *et al.* [37]	Platinum-based	✗	Prognostic	✓
Gillet *et al.* [38]	NS	✗	Prognostic	✓
Chao *et al.* [39]	NS	✗	Prognostic	✗
Schlumbrecht *et al.* [40]	Platinum/Taxane		Prognostic	✗
Glaysher *et al.* [41]	Platinum, Platinum/Paclitaxel	✓	Predictive	✓
Yan *et al.* [42]	Platinum-based	✗	Prognostic	✗
Yoshihara *et al.* [43]	Platinum/Taxane		Prognostic	✓
Williams *et al.* [44]	NS	✓	Predictive	✓
Denkert *et al.* [45]	Carboplatin/Paclitaxel		Prognostic	✓
Matsumura *et al.* [46]	Platinum-based	✓	Predictive	✓
Crijns *et al.* [47]	Platinum, Platinum/			
	Cyclophosphamide, Platinum/Paclitaxel	✓	Prognostic	✓
Mendiola *et al.* [48]	Platinum/Taxane		Prognostic	✓
Gevaert *et al.* [49]	NS	✗	Prognostic	✓
Bachvarov *et al.* [50]	Carboplatin/Paclitaxel,			
	Carboplatin/Cyclophosphamide, Cisplatin/Paclitaxel	✗	Prognostic	✓
Netinatsunthorn *et al.* [51]	Platinum/Cyclophosphamide		Prognostic	✗
De Smet *et al.* [52]	Platinum/Cyclophosphamide, Platinum/Paclitaxel	✗	Prognostic	✓
Helleman *et al.* [53]	Platinum/Cyclophosphamide, Platinum-based	✗	Prognostic	✓
Spentzos *et al.* [54]	Platinum/Taxane		Prognostic	✓
Jazaeri *et al.* [55]	Carboplatin/Paclitaxel, Cisplatin/Cyclophosphamide, Carboplatin/Docetaxel, Carboplatin	✗	Prognostic	✓
Raspollini *et al.* [56]	Cisplatin/Cyclophosphamide, Carboplatin/Cyclophosphamide, Carboplatin/Paclitaxel	✗	Prognostic	✗
Hartmann *et al.* [57]	Cisplatin/Paclitaxel, Carboplatin/Paclitaxel	✗	Prognostic	✓
Spentzos *et al.* [58]	Platinum/Taxane		Prognostic	✓
Selvanayagam *et al.* [59]	Cisplatin/Cyclophosphamide, Carboplatin/Cyclophosphamide, Cisplatin/Paclitaxel	✗	Prognostic	✓
Iba *et al.* [60]	Carboplatin/Paclitaxel		Prognostic	✗
Kamazawa *et al.* [61]	Carboplatin/Paclitaxel		Prognostic	✗
Vogt *et al.* [62]	Etoposide, Paclitaxel/Epirubicin, Carboplatin/Paclitaxel	✓	Predictive	✗

If more than one value is given, the study included patients treated with different treatments. NS: Not Specified.

**Table 6:** **Basic modelling information of papers included in systematic review**

Study	Prediction	Prediction method	Predictive ability
Jeong *et al.* [22]	Overall Survival	Student’s T test, Hierarchical clustering, Compound covariate predictor algorithm, Cox proportional hazards regression, Kaplan-Meier curves, Log-rank test, ROC analysis	‘Taxane-based treatment significantly affected OS for patients in the YA subgroup (3 year rate: 74.4% with taxane vs. 37.9% without taxane, p=0.005 by log-rank test)’, ‘estimated hazard ratio for death after taxane-based treatment in the YA subgroup was 0.5 (95% *CI*=0.31−−0.82,*p*=0.005)’
Lisowska *et al.* [23]	Chemoresponse, Disease-Free Survival, Overall Survival	Support vector machines, Kaplan-Meier curves, Log-rank test	No genes found to be significant in the training set were significant in the test set, for chemoresponse, DFS or OS
Roque *et al.* [24]	Overall Survival	Kaplan-Meier curves, Log-rank test, Student’s T test	‘OS was predicted by increased class III *β*-tubulin staining by both tumor (*HR*3.66, 96*%CI*=1.11–12.1, *p*=0.03) and stroma (*HR*4.53, 95*%CI*=1.28–16.1, *p*=0.02)’
Li *et al.* [3]	Chemoresponse (chemoresistant vs. chemosensitive)	Correlation of p-CFL1 staining and chemoresponse	‘immunostaining of p-CFL1 was positive in 77.3*%* of chemosensitive and in 95.9*%* of the chemoresistant’ (*p*=0.014, *U*=157.5)
Schwede *et al.* [25]	Stem cell-like subtype, Disease-Free Survival, Overall Survival	ISIS unsupervised bipartitioning, Diagonal linear discriminant analysis, Gaussian mixture modelling, Kaplan-Meier curves, Log-rank test	OS (p values): Dressman =0.0354, Crijns =0.021, Tothill =4.4*E*−7
Verhaak *et al.* [26]	Poor Prognosis vs. Good Prognosis	Significance analysis of microarrays, Single sample gene set enrichment analysis, Kaplan-Meier curves, Log-rank test	Good or Poor prognosis, likelihood ratio =44.63
Obermayr *et al.* [27]	Disease-Free Survival, Overall Survival	Kaplan-Meier curves, Cox proportional hazards regression, *χ*^2^ test	‘The presence of CTCs six months after completion of the adjuvant chemotherapy indicated relapse within the following six months with 41*%* sensitivity, and relapse within the entire observation period with 22*%* sensitivity (85*%* specificity)’
Han *et al.* [28]	Complete Response or Progressive Disease	Supervised principal component method	349 gene signature: ROC AUC =0.702, *p*=0.022. 18 gene: ROC AUC =0.614, *p*=0.197.
Hsu *et al.* [29]	Progression-Dree Survival	Semi-supervised hierarchical clustering	Good Response vs. Poor Response, *p*=0.021
Lui *et al.* [30]	Chemosensitivity, Overall Survival, Progression-Dree Survival	Predictive score using weighted voting algorithm, Kaplan-Meier curves, Log-rank Test, Cox proportional hazards regression	Response of 26 of 35 patients in an independent data set was correctly predicted, patients in the low-scoring group exhibited poorer PFS (*HR*=0.43, *p*=0.04), ROC AUC = 0.90(0.86–0.95)
Kang *et al.* [31]	Overall Survival, Progression-Free Survival, Recurrence-Free Survival	Kaplan-Meier curves, Log-rank test, Cox proportional hazards regression, Pearson correlation coefficient	Berchuck dataset: *HR*=0.33, 95*%CI*=0.13–0.86, *p*=0.013; Tothill dataset: *HR*=0.61, 95*%CI*=0.36–0.99, *p*=0.044
Gillet *et al.* [32]	Overall Survival, Progression-Free Survival	Supervised principle components method, Cox proportional hazards regression, Kaplan-Meier curves, Log-rank test	‘An 11-gene signature whose measured expression significantly improves the power of the covariates to predict poor survival’(*p*<0.003)
Ferriss *et al.* [33]	Overall Survival	COXEN coefficient, Mann-Whitney U test, ROC analysis, Unsupervised Hierarchical Clustering	Carboplatin: sensitivity = 0.906, specificity = 0.174, PPV = 60*%*, NPV = 57*%* (UVA-55 validation set)
Brun *et al.* [34]	2-year Disease-Free Survival	Student’s T test, Principal component analysis, Concordance index, Kaplen-Meier curves, Log-rank test	No genes were found to have prognostic value
Skirnisdottir and Seidal [35]	Recurrence, Disease-Free Survival	*χ*^2^ test, Kaplan-Meier curves, Log-rank test, Logistic regression, Cox proportional hazards regression	p53-status (*OR*=4.123, *p*=0.009; *HR*=2.447, *p*=0.019) was a significant and independent factor for tumor recurrence and DFS.
Brenne *et al.* [36]	OC or MM, Progression-Free Survival, Overall Survival	Mann-Whitney U test, Kaplan-Meier curves, Log-rank test, Cox proportional hazards regression	Cox Multivariate Analysis: EHF mRNA expression in pre-chemotherapy effusions was an independent predictor of PFS (*p*=0.033, relative risk=4.528)
Sabatier *et al.* [37]	Progression-Free Survival, Overall Survival	Cox proportional hazards regression, Pearson’s coefficient correlation score	Favourable vs. Unfavourable: ‘sensitivity = 61.6*%*, specificity = 62.4*%*, *OR*=2.7, 95*%CI*=1.7–4.2; *p*=6.1×10^−06^, Fisher’s exact test’
Gillet *et al.* [38]	Overall Survival, Progression-Free Survival, Treatment Response	Linear regression, Hierarchical clustering, Kaplan-Meier curves, Log-rank test	‘6 gene signature alone can effectively predict the progression-free survival of women with ovarian serous carcinoma (log-rank *p*=0.002)’
Chao *et al.* [39]	Chemoresistance	Interaction and expression networks for pathway identification, pathway intersections, betweenness and degree centrality, Student’s T test	No statistical measure available. Many genes identified have previously been found experimentally
Schlumbrecht *et al.* [40]	Overall Survival, Recurrence-Free Survival	Linear regression, Logistic regression, Cox proportional hazards regression, Kaplan-Meier curves, Unsupervised cluster analysis, Log-rank test, Mann-Whitney U test, *χ*^2^ test	‘Greater EIG121 expression was associated with shorter time to recurrence (*HR*=1.13 (*CI*=1.02–1.26), *p*=0.021)’, ‘Increased expression of EIG121 demonstrated a statistically significant association with worse OS (*HR*=1.21 (*CI*1.09–1.35), *p*<0.001)’
Glaysher *et al.* [41]	Chemosensitivity	AIC gene selection, Multiple linear regression	Cisplatin: $R^{2}_{\textit {adj}} = 0.836$, *p*<0.001
Yan *et al.* [42]	Chemosensitivity	ANOVA, Student’s T test, Mann-Whitney U test	‘Immunostaining scores [Annexin A3] are significantly higher in platinum-resistant tumors (*p*=0.035)’
Yoshihara *et al.* [43]	Progression-Free Survival	Cox proportional hazards regression, Ridge regression, Prognostic index, ROC analysis, Kaplan-Meier curves, Log-rank test	‘Prognostic index was an independent prognostic factor for PFS time (*HR*=1.64, *p*=0.0001)’, sensitivity = 64.4*%*, specificity = 69.2*%*
Williams *et al.* [44]	Overall Survival	COXEN score, Kaplan-Meier curves, Student’s T test, ROC analysis, Spearman’s rank correlation coefficient, Logistic regression, Log-rank test	Carboplatin and Taxol: sensitivity = 77*%*, specificity = 56*%*, *PPV*=71*%*, *NPV*=78*%*
Denkert *et al.* [45]	Overall Survival	Semi-supervised analysis via Cox scoring, Principal components analysis, Kaplan-Meier curves, Log-rank test, Cox proportional hazards regression	Duke *et al*.: ‘clinical outcome is significantly different depending on the OPI (*p*=0.021), with an HR of 1.7 (CI 1.1–2.6)’
Matsumura *et al.* [46]	Taxane sensitivity, Overall Survival	Hierarchical clustering, Kaplan-Meier curves, Log-rank test	‘Patients in the YY1-High cluster who were treated with paclitaxel showed improved survival compared with the other groups (*p*=0.010)’
Crijns *et al.* [47]	Overall Survival	Supervised principal components method, Cox proportional hazards regression, Kaplan-Meier curves, Log-rank test, *χ*^2^ test	OSP: (High-risk vs. low-risk) *HR*=1.940, *CI*=1.190–3.163, *p*=0.008
Mendiola *et al.* [48]	Progression-Free Survival, Overall Survival	Kaplan-Meier curves, Log-rank test, AIC-based model selection, ROC curves, Cox proportional hazards regression	OS: sensitivity = 87.2*%*, specificity = 86.4*%*
Gevaert *et al.* [49]	Platin Resistance/Sensitivity, Stage	Principal component analysis, Least squares support vector machines	Platin-Resistance/Sensitivity: sensitivity = 67*%*, specificity = 40*%*, accuracy = 51.11*%*
Bachvarov *et al.* [50]	Chemoresistance	Hierarchical Clustering, Support vector machines	No prediction metric applied
Netinatsunthorn *et al.* [51]	Overall Survival, Recurrence-Free Survival	Kaplan-Meier curves, Cox proportional hazards regression	OS: *HR*=1.98, 95*%CI*=1.28–3.79, *p*=0.0138 ; RFS: *HR*=3.36, 95*%CI*=1.60–7.03, *p*=0.0017
De Smet *et al.* [52]	Stage I vs. Advanced stage, Platin-sensistive vs. Platin-resistant	Principal component analysis, Least squares support vector machines	Estimated Classification Accuracy: Stage I vs Advanced Stage =100*%*, Platin-sensitive vs. Platin-resistant =76.9*%*
Helleman *et al.* [53]	Chemoresponse (responder vs. non-responder)	Class prediction, Hierarchical clustering, Principal component analysis	Test set: *PPV*=24*%*, *NPV*=97*%*, sensitivity =89*%*, specificity =59*%*
Spentzos *et al.* [54]	Chemoresponse (pathological-CR or PD), Disease-Free survival, Overall Survival	Class prediction analysis, Compound covariate algorithm, Average linkage hierarchical clustering, Kaplan-Meier curves, Log-rank test, Cox proportional hazards regression	Cox PH (resistant vs. sensitive): Recurrence *HR*=2.7 (95*%CI*=1.2–6.1), Death *HR*=3.9 (95*%CI*=3.1–11.4)
Jazaeri *et al.* [55]	Clinical response	Class prediction	9 most significantly differentially expressed genes, primary chemoresistant vs. primary chemosensitive: accuracy =77.8*%*
Raspollini *et al.* [56]	Overall Survival (high vs. low)	Univariate logistic regression, *χ*^2^ test	COX-2: *OR*=0.23, 95*%CI*=0.06–0.77, *p*=0.017; MDR1: *OR*=0.01, 95*%CI*=0.002–0.09, *p*=<0.0005
Hartmann *et al.* [57]	Time To Relapse (early vs.late)	Support vector machine, Kaplan-Meier curves, Log-rank test, average linkage clustering	Accuracy =86*%*, *PPV*=95*%*, *NPV*=67*%*
Spentzos *et al.* [58]	Disease-Free Survival, Overall Survival	Supervised pattern recognition/class prediction, Kaplan-Meier curves, Log-rank test, Cox proportional hazards regression	Unfavourable vs. Favourable OS : (CPH) *HR*=4.6, 95*%CI*=2.0–10.7, *p*=0.0001
Selvanayagam *et al.* [59]	Chemoresistance (chemoresistant vs. chemosensitive)	Supervised voice-pattern recognition algorithm (clustering)	*PPV*=1, *NPV*=1
Iba *et al.* [60]	Chemoresponse, Overall Survival	Kaplan-Meier curves, Log-rank test, Cox propotionate hazards regression, ROC analysis, *χ*^2^ test, Student’s T test, Mann-Whitney U test	‘Patients with c-myc expression of over 200 showed a significantly better 5-year survival rate (69.8*%* vs. 43.5*%*)’, *p*<0.05
Kamazawa *et al.* [61]	Chemoresponse (CR or PR vs. NC or PD)	Defined threshold expressionto divide responders and non-responders	MDR-1 (all samples): specificity =95*%*, sensitivity = 100*%*, predictive value =96*%*
Vogt *et al.* [62]	Chemoresistance	Correlation of AUC from in-vitro ATP-CVA and gene expression	All p values for correlation of drugs and genes were >0.05

If more than one value is given, the study used multiple different prediction methods or predicted more than one endpoint.

### Tissue source

For studies involving RNA extraction the tissue source is an important consideration, as RNA degradation and fragmentation could affect the results of techniques involving amplification. This is a notable issue in formalin fixed paraffin embedded (FFPE) tissue, due to the cross-linking of genetic material and proteins [63]. Of the 42 papers included in this review, the majority used fresh-frozen biopsy tissue. The numbers of each tissue source may be found in Table 7, and the tissue source used by individual papers may be found in Table 2. Nine papers did not use an RNA source directly as secondary data was used. Data sources were mostly other studies or data repositories, such as the TCGA dataset. Two studies did not specify the source tissue though extraction and expression measurement methods were detailed.

**Table 7:** **Numbers of studies using various mRNA sources**

mRNA source	Number of studies
FFPE tissue	12
Fresh-frozen tissue	22
Fresh-frozen effusion	2
Fresh tissue	1
Blood	1
Not used	9
Not specified	2

The majority of papers in this review used fresh-frozen tissue. This choice was likely made to minimise RNA degradation and hence improve measurement accuracy. Due to the risk of RNA degradation because of long storage times and the fixing process applied to FFPE tissue, it is often expected that FFPE tissue will be irreversibly cross-linked and fragmented. However, following investigation into RNA integrity when extracted from paired FFPE and fresh-frozen tissue, Rentoft *et al.* [64] found that for most samples up- and down-regulation of four genes was found to be the same whether measured in FFPE or fresh-frozen tissue. They concluded that, if samples were screened to ensure RNA quality, FFPE material can successfully provide RNA for gene expression measurement.

The use of fresh-frozen tissue in a research setting is not unusual, as can be seen from the fact that this tissue type was most popular in this review. However, for translational research expected to lead to a clinical test, this is not as reasonable. FFPE tissue is much more readily available, due to simpler acquisition and storage, and tissue is already taken for histological analysis. Therefore a model capable of using data obtained from FFPE tissue is much more likely to be applicable in a clinical setting.

Another important consideration is the proportion of neoplastic cells in the sample. For each paper the reported proportion may be seen in Table 2. Of the 42 papers, 14 reported that the proportion of cancerous cells was measured. This was usually done using hematoxylin and eosin stained histologic slides. It is important for the gene expression measurement that the tissue used contains a high proportion of neoplastic cells, and hence it is important that this pre-analytical variable is controlled. Of the studies in this review, those reporting the percentage cancerous cells were evenly distributed between FFPE and fresh-frozen tissues.

### Gene or protein expression quantification

Of the studies highlighted by this review, there were four main techniques applied for gene or protein expression measurement: Probe-target hybridization microarrays, quantitative PCR, reverse transcription end-point-PCR, and immunohistochemical staining. Of these methods only immunohistochemistry measures protein expression, via classification of the level of staining, and the other methods quantify gene expression via measurement of mRNA copy number.

Methods involving probe-target hybridization are available commercially, and 19 of the 42 studies utilised these. For example the Affymetrix^®;^ Human U133A 2.0 GeneChip and the Agilent^®;^ Whole Human Genome Oligo Microarray were both used by multiple studies. Additionally, 7 studies used custom-made probe-target hybridization arrays. Probe-target hybridisation arrays generally measure thousands of genes and hence can provide a wealth data per sample. TaqMan^®;^ microfluidic arrays or quantitative-PCR were used by 16 studies. These techniques are typically used for smaller panels of genes. The TaqMan^®;^ arrays for example may contain up to 384 genes per array. These methods are more targeted and hence the price per sample is usually lower.

Immunohistochemistry is a more labour-intensive technique, requiring staining for each gene considered, and hence was mostly only used by studies using small numbers of genes. This technique, which is semi-quantitative due to the scoring systems employed, also suffers from a lack of standardisation of procedures. Of the 11 papers using this technique, the maximum number of genes analysed was seven, and the mean number of genes assessed was 2.8. Although these studies provide useful information regarding the correlation of particular genes with outcome, the small numbers of genes is likely to result in an incomplete gene signature and low predictive power.

Several of the papers utilising quantifiable techniques used an alternative method or replicates to obtain a measure of the assay variability. Five papers involving commercial or custom microarrays also used reverse transcription PCR (RT-PCR) to measure the expression of a small number of genes for comparison and one study used samples run in duplicate to calculate the coefficient of variation. Of the studies using TaqMan microfluidic arrays, two used samples run in duplicate to obtain the coefficient of variation. However, even fewer papers reported a metric representing the level of variability found. Two studies reported a coefficient of variation; Glaysher *et al.* [41] reported CoV=2*%*=0.02 for TaqMan arrays and Hartmann *et al.* [57] reported CoV=0.2 for their custom microarray. Another two reported Spearman’s or Pearson’s r coefficients of correlation between microarray and RT-PCR results. Yoshihara *et al.* [43] gave Pearson r values ranging from 0.5 to 0.8, and Crijns *et al.* [47] gave Spearman’s r values between -0.6 and -0.9.

### Histology

Table 4 details the histology (types and stages) of the patient samples used by each study. As may be seen, the majority of studies were heterogeneous with respect to the types of cancer included. However, 23 of the 42 studies used at least 80% serous samples, suggesting that the majority of information contributed to the gene signatures of these studies is related to the mechanisms and pathways in serous cancer. In the authors’ opinion it is important to identify the histologies of patient samples: although treatment is currently the same across types, response to chemotherapy has been found to vary [9,65,66]. It therefore may be advisable for future studies to include histological information when developing models predicting chemotherapy response.

### Chemotherapy

Table 5 lists the chemotherapy treatments undergone by patients in each study. The 10 papers labelled NS did not specify the regimen applied, though the patients did have chemotherapy. These cohorts cannot therefore be assumed to be homogeneous with respect to patient chemotherapy treatment. All studies that specified the chemotherapy regimen undergone by patients noted at least one platinum-based treatment. Of these, 24 included patients treated with a platinum-taxane combination and 10 with a cyclophosphamide-platinum combination. It is important to note that 19 of the 42 papers stated the population was heterogeneous with regards to chemotherapy treatments and, of those that did, only 8 included patient treatment history as a feature of the study. The aims of the majority of the studies were to identify genes of which the expression may be used to predict survival time, or prognosis. As already noted, the presence of resistance to the chemotherapy agent administered will dramatically affect the survival of a patient. It is therefore reasonable to expect the gene signatures identified to include genes responsible for chemoresistance, which will depend on the mechanism of action of the drug. Using a heterogeneous cohort in terms of chemotherapy treatment may then be causing problems with the identification of a minimal predictive gene set.

### End-point to be predicted

As may be expected, there was variation between the end-point chosen by studies for prediction. Popular end-points include overall survival, progression-free survival and response to chemotherapy. The endpoints considered by each study may be found in Table 6. Of these some are clinical endpoints, such as overall survival, others use non-clinical endpoints, such as response to chemotherapy, many of which are considered to be surrogates for overall survival. For cancer studies, overall survival is considered to be the most reliable and is the variable that is of most interest when considering the effect of an intervention.

### Model development

Within this review, many different modelling techniques were used to identify an explanatory gene signature to predict patient outcome. The most popular was Cox proportional hazards regression, which was applied by 17 studies. This was closely followed by hierarchical clustering, which was used by 11 studies. All other methods were used by 8 or fewer studies. In total 24 different types of modelling techniques were applied, ranging from statistical tests such as Student’s T test and Mann-Whitney U test, to logistic regression, to ridge regression. Table 8 lists the modelling techniques identified and the number of studies that employed them. It is of interest that most of the techniques applied are forms of classification. These methods result in samples being assigned to groups, such as ‘good prognosis’ and ‘poor prognosis’. Whilst this may be useful in some settings, for a clinically-applicable tool a regression technique may be more appropriate as it will provide a value, such as a likelihood of relapse, rather than simply a class. Techniques in Table 8 capable of a numeric prediction include logistic and linear regression, Cox proportional hazards regression, and ridge regression.

**Table 8:** **Key modelling techniques applied by studies in the review**

Technique	Number of papers
Cox proportional hazards regression	17
Hierarchical clustering	11
Principal components analysis	8
Student’s T test	7
Scoring algorithm	6
Support Vector Machines	5
Correlation coefficients	5
Mann-Whitney U test	5
*χ*^2^ test	5
ROC analysis	5
Class prediction	4
Logistic regression	3
Linear regression	3
AIC gene selection	2
Concordance index	1
Pathway interaction networks	1
ANOVA	1
Expression threshold identified	1
Gene set enrichment analysis	1
Linear discriminant analysis	1
ISIS bipartitoning	1
Gaussian mixture modelling	1
Significance analysis of microarrays	1
Ridge regression	1

Jointly with the modelling methods identified above, 23 of the 42 studies implemented Kaplan-Meier curves to visualise the survival of the patient classes identified by the models. This enables the difference in survival between classes, for example ‘good prognosis’ and ‘poor prognosis’, to be seen and assessed. The application of a log-rank test assesses the separation of the curves and identifies whether there is a statistically significant difference in survival distribution between the classes. It should be noted that, although this gives an idea of separation of classes achieved by the model, the model results must still be compared with known outcomes to check positive and negative predictive power. This step was missing in several papers, such as Gillet *et al.* [38], where the p value returned by the log-rank test is given as the measure of model success.

It is important to highlight the difference between prognostic and predictive models. A prognostic model is one capable of predicting prognosis, such as survival time, using patient information and biomarkers and does not vary between different treatment options. In contrast, a predictive model is one able to predict the effect of a treatment on patient prognosis [67,68]. It is therefore clear that, although prognostic models may be useful for research purposes and when one treatment option is available (such as the standard platinum-taxane combination), predictive models have a much greater part to play in stratified medicine where the aim is to identify the most appropriate treatment on a patient-by-patient basis. In order for a model to be predictive, the effects of multiple treatments must be considered and the response compared with the biomarker status. Classification of the studies as prognostic or predictive may be seen in Table 5. Of the papers identified by this review, only a minority considered the effects of chemotherapy treatment on the predicted outcome and hence could be considered predictive. Glaysher *et al.* [41] and Vogt *et al.* [62] produced separate models for various treatments, allowing the effects of different drugs and combinations to be compared. Both studies applied drugs *in vitro* to cultured tissue to measure response to chemotherapy. This was combined with gene expression measurements to form the model training data set. In this way the same patient samples may be used to create a set of models predicting response to a variety of drugs. These models are therefore predictive rather than prognostic. Alternatively, models may be trained on sets of patients split by treatments undergone, which would lead to treatment-specific models predicting response to the particular drug. This method was used by Jeong *et al.* [22], Ferriss *et al.* [33], Williams *et al.* [44] and Matsumura *et al.* [46]. Additionally, the use of a model variable specifying patient treatment history could allow these models to be combined onto one using a single training set of all patients. The model may then be passed a variable specifying the drug of interest for resistance prediction. A simple version of this method was implemented by Crijns *et al.* [47], who included a feature for whether a patient was treated with paclitaxel. It is clear that the integration of patient chemotherapy treatment into these models is underused, and it is likely to be beneficial for this to be incorporated into future research.

### Genes identified

Of the 42 papers in this review, 32 provided full or partial lists of the genes identified by their models. Of the remainder, it was common that the gene sets were large or that the genes were not explicitly identified by the model, as is the case with modelling techniques such as principal components analysis.

In total across the papers, 1298 unique genes were selected by models and of these 93.53*%* were found by only one paper. The most commonly chosen gene was selected by only four papers. Table 9 shows the numbers and percentages of genes chosen by one to four papers.

**Table 9:** **Numbers and percentages of genes featured in the gene sets of various numbers of papers**

Number of papers	Number of genes	Percent of genes
identifying a gene		
1	1214	93.53*%*
2	78	6.01*%*
3	5	0.385*%*
4	1	0.08*%*

A list of the genes identified by the papers in the review may be found in Table 10.

**Table 10:** **List of genes reported by studies included in this review**

A1BG	CHPF2	FSCN1	LRRC16B	PKD1	SOBP
A2M	CHRDL1	FXYD6	LRRC17	PKHD1	SORBS3
AADAC	CHRNE	FZD4	LRRC59	PLA2G7	SOS1
AAK1	CHST6	FZD5	LRSAM1	PLAA	SOX12
ABCA13	CHTOP	G0S2	LSAMP	PLAU	SOX21
ABCA4	CIAPIN1	G3BP1	LSM14A	PLAUR	SPANXD
ABCB1	CIB1	GABRP	LSM3	PLCB3	SPATA13
ABCB10	CIB2	GAD1	LSM7	PLEC	SPATA18
ABCB11	CIITA	GALNT10	LSM8	PLEK	SPATA4
ABCB7	CILP	GAP43	LTA4H	PLIN2	SPC25
ABCC3	CITED2	GART	LTB	PLS1	SPDEF
ABCC5	CKLF	GATAD2A	LTK	PMM1	SPEN
ABCD2	CLCA1	GCH1	LUC7L2	PMP22	SPHK2
ABCG2	CLCNKB	GCHFR	LY6K	PMVK	SPOCK2
ABLIM1	CLDN10	GCM1	LY96	PNLDC1	SPTBN2
ACADVL	CLIP1	GDF6	LZTFL1	PNLIPRP2	SRC
ACAT2	CNDP1	GFRA1	MAB21L2	PNMA5	SREBF2
ACKR2	CNKSR3	GGCT	MAD2L2	POFUT2	SRF
ACKR3	CNN2	GGT1	MAGEE2	POLH	SRRM1
ACO2	CNOT8	GJB1	MAGEF1	POLR3K	SRSF3
ACOT13	CNTFR	GLRX	MAK	POMP	SSR1
ACP1	cofilin1	GMFB	MAMLD1	POU2AF1	SSR2
ACRV1	COL10A1	GMPR	MANF	POU5F1	SSUH2
ACSM1	COL21A1	GNA11	MAP6D1	PPAP2B	SSX2IP
ACSS3	COL3A1	GNAO1	MAPK1	PPAT	ST6GALNAC1
ACTA2	COL4A4	GNAZ	MAPK1IP1L	PPCDC	STC2
ACTB	COL4A6	GNG4	MAPK3	PPCS	STK38
ACTBL3	COL6A1	GNG7	MAPK8IP3	PPFIA3	STX12
ACTG2	COL7A1	GNL2	MAPK9	PPIC	STX1B
ACTR3B	COX8A	GNMT	MAPKAP1	PPIE	STX7
ACTR6	CPD	GNPDA1	MAPKAPK2	PPP1R1A	STXBP2
ADAMDEC1	CPE	GOLPH3	MARCKS	PPP1R1B	STXBP6
ADAMTS5	CPEB1	GPIHBP1	MARK4	PPP1R2	SUB1
ADIPOR2	CRCT1	GPM6B	MATK	PPP1R26	SULT1C2
ADK	CREB5	GPR137	MB	PPP2R3C	SULT2B1
AEBP1	CRYAB	GPT2	MBOAT7	PPP2R5C	SUPT5H
AF050199	CRYBB1	GPX2	MCF2L	PPP2R5D	SUSD4
AF052172	CRYL1	GPX3	MCL1	PPP4R4	SUV420H1
AFM	CRYM	GPX8	MCM3	PPP6R1	SV2C
AFTPH	CSE1L	GRAMD1B	MDC1	PRAP1	SYNM
AGFG1	CSPP1	GRB2	MDFI	PRELP	SYT1
**AGR2**	CSRP1	GRK6	MDK	PRKAB1	SYT11
AGT	CSRP3	GRM2	MDR-1	PRKCH	SYT13
AIPL1	CST6	GRPEL1	MEA1	PRKCI	TAC3
**AKAP12**	CST9L	GRSF1	MEAF6	PRKD3	TAP1
AKR1A1	CT45A6	GSPT1	MECOM	PROC	TASP1
AKR1C1	CTA-246H3.1	GSTM2	MEF2B	PROK1	TBCC
AKT1	CTNNBL1	GSTT1	MEGF11	PRPF31	TBP
AKT2	CTSD	GTF2E1	MEST	PRRX1	TCF15
ALCAM	CUTA	GTF2F2	METRN	PRSS16	TCF7L2
ALDH5A1	CX3CL1	GTF2H5	METTL13	PRSS22	TENM3
ALDH9A1	CXCL1	GTPBP4	METTL4	PRSS3	TEX30
ALG5	CXCL10	GUCY1B3	MFAP2	PRSS36	TFF1
ALMS1	CXCL12	GYG1	MFSD7	PSAT1	TFF3
AMPD1	CXCL13	GYPC	MGMT	PSMB5	TFPI2
ANKHD1	CXCR4	GZMB	MINOS1	PSMB9	TGFB1
ANKRD27	CYB5B	GZMK	MKRN1	PSMC4	THBS4
ANXA3	CYBRD1	H2AFX	MLF2	PSMD1	TIAM1
ANXA4	CYP27A1	H3F3A	MLH1	PSMD12	TIMM10B
AOC1	CYP2E1	HAP1	MLX	PSMD14	TIMM17B
AP2A2	CYP3A7	HBG2	MMP1	PSME4	TIMP1
APC	CYP4X1	HDAC1	MMP10	PTBP1	TIMP2
API5	CYP4Z1	HDAC2	MMP12	PTCH2	TIMP3
APOE	CYP51A1	HECTD4	MMP13	PTEN	TKTL1
AQP10	CYSTM1	HES1	MMP16	PTGDS	TLE2
AQP5	CYTH3	HEY1	MMP17	PTGS2	TM9SF2
AQP6	D4S234E	HHIPL2	MMP3	PTP4A1	TM9SF3
AQP9	DAP	HIF1A	MMP7	PTP4A2	TMCC1
ARAF	DAPL1	HIP1R	MMP9	PTPRN2	TMED5
ARAP1	DBI	HIPK1	MPZL1	PTPRS	TMEM139
AREG	DCBLD2	HIST1H1C	MRPL2	PWP2	TMEM14B
ARFGEF2	DCHS1	HK2	MRPL35	QPRT	TMEM150A
ARHGAP29	DCK	HLAA	MRPL49	R3HDM2	TMEM161A
ARHGDIA	DCTN5	HLADMB	MRPS12	RAB26	TMEM259
ARL14	DCTPP1	HLADOB	MRPS17	RAB27B	TMEM260
ARL6IP4	DCUN1D4	HMBOX1	MRPS24	RAB40B	TMEM45A
ARMC1	DCUN1D5	HMGCS1	MRPS9	RAB5B	TMEM50A
ARNT2	DDB1	HMGCS2	MRS2	RAB5C	TMPRSS3
ARPC4	DDB2	HMGN1	MSH2	RABIF	TMSB15B
ASAP1	DDR1	HMOX2	MSL1	RAC1	TMTC1
ASAP3	DDX23	HNRNPA1	MSMO1	RAC3	TMX2
ASF1A	DDX49	HNRNPUL2	MST1	RAD23A	TNFRSF17
ASIP	DEFB132	HOPX	MT1G	RAD51	TNS1
ASPA	DERL1	HOXA5	MTCP1	RAD51AP1	TOMM40
ASPHD1	DFNB31	HOXB6	MTMR11	RANBP1	TONSL
ASS1	DHCR7	HPN	MTMR2	RANGAP1	TOP1
ASUN	DHRS11	HRASLS	MTPAP	RARRES2	**TOP2A**
ATM	DHRS9	Hs.120332	MTUS1	RB1	TOX3
ATP1B3	DHX15	HS3ST1	MTX1	RBBP7	**TP53**
ATP5D	DHX29	HS3ST5	MUS81	RBFA	TP53TG5
ATP5F1	DIAPH3	HSD11B2	**MUTYH**	RBM11	TP73
ATP5L	DICER1	HSD17B11	MXD1	RBM39	TPD52
ATP6V0E1	DIRC1	HSPA1L	MXI1	RCHY1	TPM2
ATP7B	DKK1	HSPA4	MYBPC1	RER1	TPP2
ATP8A2	DLAT	HSPA8	MYC	RFC3	TPPP
AUP1	DLEU2	HSPB7	MYCBP	RGL2	TPRKB
AURKA	DLG1	HSPD1	MYL9	RGP1	TRA
AURKC	DLG3	HTATIP2	MYO1D	RGS19	TRAF3IP2
AVIL	DLGAP4	HTN1	MYOM1	RHOT1	TRAM1
B3GALNT1	DLGAP5	HTR3A	NANOS1	RHPN2	TRAPPC4
B3GNT2	DMRT3	ICAM1	NASP	RIIAD1	TRAPPC9
B4GALT5	DNAH2	ICAM5	NBEA	RIN1	TREML1
BAG3	DNAH7	ID1	NBL1	RIT1	TREML2
BAIAP2L1	DNAJB12	ID4	NBN	RNF10	TRIAP1
BAK1	DNAJB5	IDI1	NCAM1	RNF13	TRIM27
BASP1	DNAJC16	IFIT1	NCAPD2	RNF14	TRIM49
BAX	DNASE1L3	IGF1R	NCAPG	RNF148	TRIM58
BCHE	DOCK3	IGFBP2	NCAPH	RNF34	TRIML2
BCL2A1	DPH2	IGFBP5	NCKAP5	RNF6	TRIT1
BCL2L11	DPM1	IGHM	NCOA1	RNF7	TRMT1L
BCL2L12	DPP7	IGKC	NCOR2	RNF8	TRO
BCR-ABL	DPYSL2	IGKV1-5	NCR2	RNGTT	TRPV4
BEAN	DRD4	IHH	NCSTN	RNPEPL1	TRPV6
BEST4	DTYMK	IKZF4	NDRG2	ROBO1	TSPAN3
BFSP1	DUSP2	IL11RA	NDST1	ROR1	TSPAN4
BFSP2	DUSP4	IL15	NDUFA12	ROR2	TSPAN6
BGN	DUX3	IL17RB	NDUFA9	RP13-347D8.3	TSPAN7
BHLHE40	DYNLT1	IL1B	NDUFAB1	RP13-36C9.6	TSR1
BIN1	DYRK3	IL23A	NDUFAF4	RPA3	TTC31
BIRC5	E2F2	IL27	NDUFB4	RPL23	TTLL6
BIRC6	ECH1	IL6	NDUFS5	RPL29P17	TTPAL
BLCAP	EDF1	IL8	NEBL	RPL31	TTYH1
BLMH	EDN1	IMPA2	NETO2	RPL36	TUBB3
BMP8B	EDNRA	ING3	NEUROD2	RPP30	TUBB4A
BMPR1A	EDNRB	INHBA	NFE2	RPS15	TUBB4Q
BNIP3	EEF1A2	INPP5A	NFE2L3	RPS16	TUSC3
BOLA3	EFCAB14	INPP5B	NFIB	RPS19BP1	UBD
BPTF	EFEMP2	INSR	NFKBIB	RPS24	UBE2I
BRCA1	EFNB2	INTS12	NFS1	RPS28	UBE2K
BRCA2	EGF	INTS9	NID1	RPS4Y1	UBE2L3
BRSK1	EGFR	IRF2BP1	NIT1	RPS6KA2	UBE4B
BTN3A3	EHD1	ISCA1	NKIRAS2	RPSA	UBR5
BTNL9	EHF	ISG20	NKX31	RRAGC	UGT2B17
C11orf16	EI24	ITGAE	NKX62	RRBP1	UGT8
C11orf74	EIF1	ITGB2	NLGN1	RRN3	UHRF1BP1
C12orf5	EIF2AK2	ITGB6	NOP5/58	RSL24D1	UMOD
C16orf89	EIF3K	ITGB7	NOS3	RSU1	UPK1A
C17orf45	EIF4E2	ITLN1	NOTCH4	RTN4R	UPK1B
C17orf53	EIF5	ITM2A	NOV	RXRB	UQCRC2
C17orf70	ELF3	ITM2C	NOX1	RYBP	URI1
C1orf109	ELF5	ITPR2	NPAS3	RYR3	USP14
C1orf115	EML4	ITPRIP	NPR1	S100A10	USP18
C1orf159	ENC1	JAG2	NPR3	S100A4	USP21
C1orf198	ENOPH1	JAK2	NPTX2	S100P	UST
C1orf27	ENSA	JAKMIP2	NPTXR	SAMD4B	UTP11L
C1orf68	ENTPD4	KCNB1	NPY	SASH1	UTP20
C1QTNF3	EPB41L4A	KCNE3	NRBP2	SCAMP3	UVRAG
C20orf199	EPCAM	KCNH2	NRG4	SCARF1	VDR
C2orf72	EPHB2	KCNJ16	NRP1	SCG2	VEGFA
C4A	EPHB3	KCNN1	NSFL1C	SCGB1C1	VEGFB
C4BPA	EPHB4	KCNN3	NSL1	SCGB3A1	VEZF1
C6orf120	EPOR	KCTD1	NSMCE4A	SCNM1	VPS39
C6orf124	ERBB3	KCTD5	NT5C3A	SCO2	VPS52
C9orf3	ERCC8	KDELC1	NTAN1	SCUBE2	VPS72
C9orf47	ERMP1	KDELR1	NTF4	SDF2L1	VTCN1
CA13	ESF1	KDELR2	NUDT21	SEC14L2	VTI1B
CACNA1B	ESM1	KDM4A	NUDT9	SELT	WBP2
CACNG6	ESR1	Ki67	NUS1	SEMA3A	WBP4
CADM1	ESRP2	KIAA0125	OAS3	SENP3	WDR12
CALML3	ESYT1	KIAA0141	OASL	SENP6	WDR45B
CAMK2B	ETS1	KIAA0226	ODF4	SEPN1	WDR7
CAMK2N1	ETV1	KIAA0368	OGFOD3	SERPINB6	WDR77
CANX	EVA1A	KIAA1009	OGN	SERPIND1	WIT1
CAP1	EXOC6B	KIAA1033	OPA3	SERPINF1	WIZ
CAP2	EXTL1	KIAA1324	OR10A3	SERTAD4	WNK4
CAPN13	EYA2	KIAA1551	OR2AG1	SETBP1	WNT16
CAPN5	F2R	KIAA2022	OR4C15	SF3A3	WT1
CASC3	FAAH	KIAA4146	OR51B5	SF3B4	WTAP
CASP9	FABP1	KIF3A	OR51I1	SGCB	WWOX
CASS4	FABP7	KIFC3	OR6F1	SGCG	XBP1
CATSPERD	FADS1	KIT	OR9G9	SGPP1	XPA
CC2D1A	FADS2	KLF12	OSGEPL1	SH3PXD2A	XPO4
CCBL1	FAM133A	KLF5	OSGIN2	SHFM1	XYLT1
CCDC130	FAM135A	KLHDC3	OSM	SHOX	Y09846
CCDC135	FAM155B	KLHL7	OXTR	SIDT1	YBX1
CCDC147	FAM174B	KLK10	P2RX4	SIGLEC8	YIPF3
CCDC167	FAM19A4	KLK6	PABPC4	SIRT5	YIPF6
CCDC19	FAM211B	KPNA3	PAGR1	SIRT6	YLPM1
CCDC53	FAM217B	KPNA6	PAH	SIVA1	YWHAE
CCDC9	FAM49B	KRT10	PAK4	SIX2	YWHAZ
CCL13	FAM8A1	KRT12	PALB2	SKA3	ZBTB11
CCL2	FANCB	KYNU	PARD6B	SLAMF7	ZBTB16
CCL28	FANCE	L1TD1	PAX6	SLC12A2	ZBTB8A
CCM2L	FANCF	LAMB1	PBK	SLC12A4	ZC3H13
CCNA2	FANCG	LAMTOR5	PBX2	SLC14A1	ZCCHC8
CCNG2	FANCI	LARP4	PBXIP1	SLC15A2	ZEB2
CCT6A	FARP1	LAX1	PCF11	SLC1A1	ZFHX4
CCZ1	FAS	LAYN	PCGF3	SLC1A3	ZFP91
CD34	FASLG	LBR	PCK1	SLC22A5	ZFR2
CD38	FBXL18	LCMT2	PCNA	SLC25A37	ZKSCAN7
CD44	FCGBP	LCTL	PCNXL2	SLC25A41	ZMYND11
CD46	FCGR3B	LDB1	PCOLCE	SLC25A5	ZNF106
CD70	FEN1	LDHB	PCSK6	SLC26A9	ZNF12
CD97	FEZ1	LGALS4	PDCD2	SLC27A6	ZNF124
CDC42EP4	FGF2	LGR5	PDE3A	SLC29A1	ZNF148
CDCA2	FGFBP1	LHB	PDGFA	SLC2A1	ZNF155
CDH12	FGFR1OP	LHX1	PDGFRA	SLC2A5	ZNF180
CDH19	FGFR1OP2	LIN28A	PDGFRB	SLC37A4	ZNF200
CDH3	FGFR2	LINGO1	PDP1	SLC39A2	ZNF292
CDH4	FHL2	LIPA	PDSS1	SLC4A11	ZNF337
CDH5	FILIP1	LIPC	PDZK1	SLC5A1	ZNF432
CDK17	FJX1	LIPG	PEBP1	SLC5A3	ZNF467
CDK20	FKBP11	LMO3	PEX11A	SLC5A5	ZNF48
CDK5R1	FKBP1B	LMO4	PEX6	SLC6A3	ZNF503
CDK8	FKBP7	LOC100129250	PFAS	SLC7A2	ZNF521
CDKN1A	FLII	LOC149018	PGAM1	SMAD2	ZNF569
CDY1	FLJ41501	LOC1720	PHF3	SMC4	ZNF644
CDYL2	FLNC	LOC389677	PHGDH	SMG1	ZNF71
CEACAM5	FLOT2	LOC642236	PHKA1	SMPD2	ZNF711
CEACAM6	FLT1	LOC646808	PHKA2	SNIP1	ZNF74
CEACAM7	FMN2	LOC90925	PI3	SNRPA1	ZNF76
CEP55	FMO1	LPAR6	PIC3CD	SNRPC	ZNF780B
CES1	FN1	LPCAT2	PIGC	SNRPD3	ZYG11A
CES2	**FOXA2**	LPCAT4	PIGR	SNX13	
CFI	FOXD4L2	LPHN2	PIK3CG	SNX19	
CH25H	FOXJ1	LRIG1	PIP5K1B	SNX7	
CHIT1	FOXO3	LRIT1	PITRM1	SOAT2	

Gene names have been standardised. Genes in bold were selected by more than two studies.

It is clear that the gene sets selected by the studies are very different and there is very little overlap. The genes chosen by two or more studies may be seen in Table 11. Many of these genes are known to have links to cancer, which may suggest that these genes are therefore implicated in ovarian cancer. It is possible that, although the genes selected varied, they in fact represent similar mechanisms. This could occur if there are large sets of highly covariate genes representing particular cellular processes and the genes in the signatures were simply random selections from these gene sets. The same gene being selected by multiple papers would then be unlikely, although the same information contribution would be made. It may then be more informative to assess and compare the mechanisms controlled by the genes chosen as part of the models.

**Table 11:** **Genes chosen most commonly by studies in review**

Gene symbol	Number of studies	Function	Expression links to cancer in literature
AGR2	4	Cell migration and growth	Prostate, breast, ovarian, pancreatic
MUTYH	3	Oxidative DNA damage repair	Colorectal
AKAP12	3	Subcellular compartmentation of PKA	Colorectal, lung, prostate
TP53	3	Cell cycle regulation	Breast
TOP2A	3	Required for DNA replication	Breast, prostate, ovarian
FOXA2	3	Liver-specific transcription factor	Lung, prostate
SRC	2	Regulation of cell growth	Colon, liver, lung, breast, pancreatic
SIVA1	2	Pro-apoptotic protein	Many cancers
ALDH9A1	2	Aldehyde dehydrogenase	Many cancers
LGR5	2	Associated with stem cells	Cancer stem cells
EHF	2	Epithelial differentiation and proliferation	Prostate
BAX	2	Apoptotic activator	Colon, breast, prostate, gastric, leukaemia
CES2	2	Intestine drug clearance	Colorectal
CPE	2	Synthesis of hormones and neurotransmitters	
FGFBP1	2	Cell proliferation, differentiation and migration	Colorectal, pancreatic
TUBB4A	2	Component of microtubules	
ZNF12	2	Transcription regulation	
RBM39	2	Steroid hormone receptor-mediated transcription	
RFC3	2	Required for DNA replication	
GNPDA1	2	Triggers calcium oscillations in mammalian eggs	
ANXA3	2	Regulation of cellular growth	Prostate, ovarian
NFIB	2	Activates transcription and replication	Breast
ACTR3B	2	Actin cyctoskeleton organisation	Lung
YWHAE	2	Mediates signal transduction	Lung, endometrial
CYP51A1	2	Drug metabolism and lipid synthesis	
HMGCS1	2	Cholesterol synthesis and ketogenesis	
ZMYND11	2	Transcriptional repressor	
FADS2	2	Regulates unsaturation of fatty acids	
SNX7	2	Family involved in intracellular trafficking	
ARHGDIA	2	Regulates the GDP/GTP exchange reaction of the Rho proteins	Prostate, lung,
NDST1	2	Inflammatory response	Prostate, breast
AOC1	2	Catalyses degredation of such as histamine and spermidine	
DAP	2	Positive mediator of programmed cell death	
ERCC8	2	Transcription-coupled nucleotide excision repair	
GUCY1B3	2	Catalyzes conversion of GTP to the second messenger cGMP	
HDAC1	2	Control of cell proliferation and differentiation	Prostate, breast, colorectal, gastric
HDAC2	2	Transcriptional regulation and cell cycle progression	Cervical, gastric, colorectal
IGFBP5	2	Cell proliferation, differentiation, survival, and motility	Breast
IL6	2	Transcriptional inflammatory response, B cell maturation	Many cancers
LSAMP	2	Neuronal surface glycoprotein	Osteosarcoma
MDK	2	Cell growth, migration, angiogenesis	Many cancers
MYCBP	2	Stimulates the activation of E box-dependent transcription	
S100A10	2	Transport of neurotransmitters	Colorectal, lung, breast
SLC1A3	2	Glutamate transporter	
NCOA1	2	Stimulates hormone-dependent transcription	Breast, prostate
TIAM1	2	Modulates the activity of Rho GTP-binding proteins	Many cancers
VEGFA	2	Angiogenesis, cell growth, cell migration, apoptosis	Many cancers
RPL36	2	Component of ribosomal 60S subunit	
LBR	2	Anchors lamina and heterochromatin to the nuclear membrane	
ABCB1	2	ATP-dependent drug efflux pump for xenobiotic compounds	Many cancers
FASLG	2	Required for triggering apoptosis in some cell types	Many cancers
TIMP1	2	Extracellular matrix, proliferation, apoptosis	Many cancers
FN1	2	Cell adhesion, motility, migration processes	Many cancers
TGFB1	2	Proliferation, differentiation, adhesion, migration	Prostate, breast, colon, lung, bladder
XPA	2	DNA excision repair	Many cancers
ABCB10	2	Mitochondrial ATP-binding cassette transporter	
POLH	2	Polymerase capable of replicating UV-damaged DNA for repair	
ITGAE	2	Adhesion, intestinal intraepithelial lymphocyte activation	
ZNF200	2	Zinc finger protein	
COL3A1	2	Collagen type III, occurring in most soft connective tissues	
ACKR3	2	G-protein coupled receptor	
EPHB3	2	Mediates developmental processes	Lung, colorectal
NBN	2	Double-strand DNA repair, cell cycle control	
PCF11	2	May be involved in Pol II release following polymerisation	
DFNB31	2	Sterocilia elongation, actin cystoskeletal assembly	
BRCA2	2	Double-strand DNA repair	Breast, ovarian
AADAC	2	Arylacetamide deacetylase	
CD38	2	Glucose-induced insulin secretion	Leukaemia
CHIT1	2	Involved in degradation of chitin-containing pathogens	
CXCR4	2	Receptor specific for stromal-derived-factor-1	Breast, glioma, kidney, prostate
EFNB2	2	Mediates developmental processes	
MECOM	2	Apoptosis, development, cell differentiation, proliferation	Leukaemia
FILIP1	2	Controls neocortical cell migration	Ovarian
HSPB7	2	Heat shock protein	
LRIG1	2	Regulator of signaling by receptor tyrosine kinases	Glioma
MMP1	2	Breakdown of extracellular matrix	Gastric, breast
PSAT1	2	Phosphoserine aminotransferase	
SDF2L1	2	Part of endoplasmic reticulum chaperone complex	
TCF15	2	Regulation of patterning of the mesoderm	
EPHB2	2	Contact-dependent bidirectional signaling between cells	Colorectal
ETS1	2	Involved in stem cell development, cell senescence and death	Many cancers
TRIM27	2	Male germ cell differentiation	Ovarian, endometrial, prostate
MARK4	2	Mitosis, cell cycle control	Glioma
B4GALT5	2	Biosynthesis of glycoconjugates and saccharides	

Genes listed by number of papers selecting each gene. Gene function and links to cancer obtained via cursory literature search.

### Gene set enrichment

**Figure 2:**
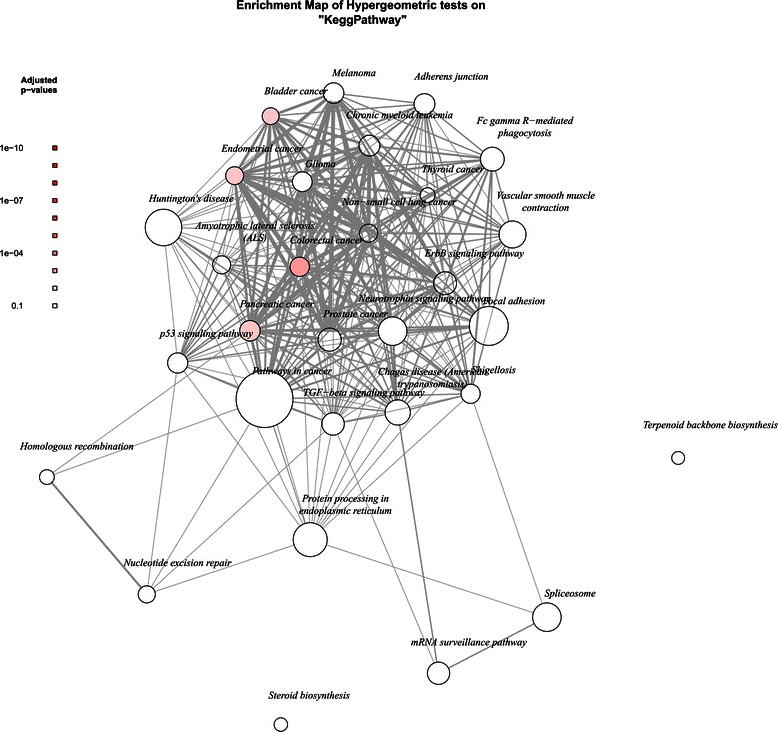
**Gene set enrichment networks for studies assessing ovarian cancer patients treated with platinum and taxane.** Network maps of the 30 most enriched KEGG pathways. Node marker size signifies the number of genes in this category, and the thickness of edges indicate the Jaccard similarity coefficient between categories. Node markers are coloured according to adjusted p value as reported by the hypergeometric test, where darker red denotes more highly significant.

**Figure 3:**
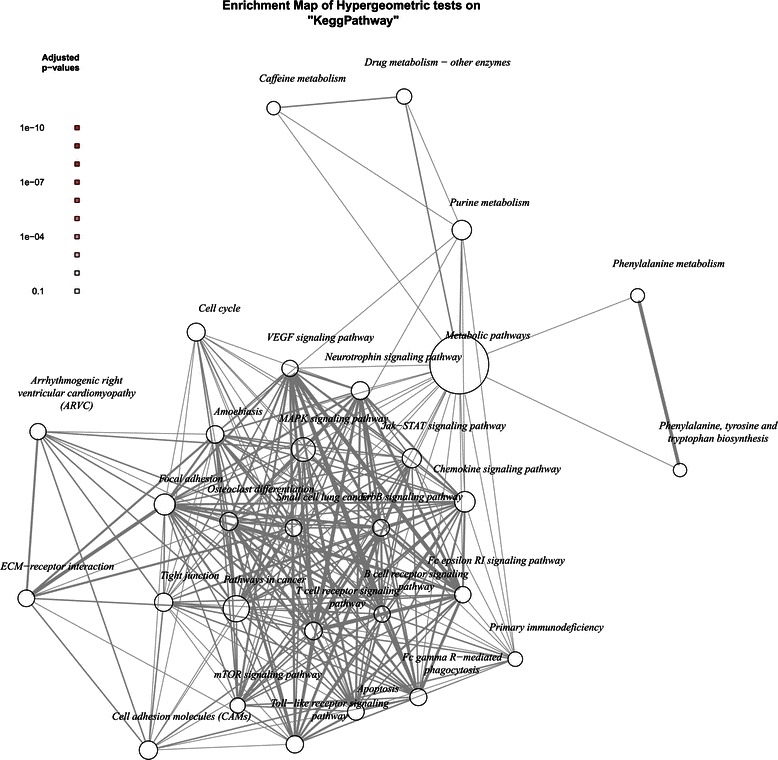
**Gene set enrichment networks for studies assessing ovarian cancer patients treated with treatments other than platinum and taxane.** Network maps of the 30 most enriched KEGG pathways. Node marker size signifies the number of genes in this category, and the thickness of edges indicate the Jaccard similarity coefficient between categories. Node markers are coloured according to adjusted p value as reported by the hypergeometric test, where darker red denotes more highly significant.

The gene sets reported by the studies identified in this review were assessed to identify whether certain biological pathways and mechanisms featured more prominently according to the genes selected. Studies were split by chemotherapy treatments recieved by the patients, and the groups identified were platinum and taxane, and other treatments (such as platinum, cyclophosphamide and combinations). Studies that did not specify the chemotherapy treatments used were excluded. Studies falling into the platinum and taxane group were Han *et al.* [28], Kang *et al.* [31], Gillet *et al.* [32], Skirnisdottir and Seidal [35], Schlumbrecht *et al.* [40], Yoshihara *et al.* [43], Denkert *et al.* [45], Hartmann *et al.* [57], Iba *et al.* [60], and Kamazawa *et al.* [61]. Studies falling into the other treatments group were Obermayr *et al.* [27], Sabatier *et al.* [27], Yan *et al.* [42], Netinatsunthorn *et al.* [51], and Helleman *et al.* [53]. The results of the gene set enrichment using the KEGG system may be seen in Figures 2 and 3. From the plots, it may be seen that both groups identify several cancer-related pathways relevant to the drug mechanisms of action.

It is informative to consider the KEGG terms in the context of the mechanisms of action of the chemotherapy drugs applied. Both groups contain patients treated with platinum single agents or platinum-containing combinations. It should therefore be expected that processes associated with the mechanism of action of platinum will be enriched. Once activated, the platinum binds to DNA and results in the formation of monoadducts, intra-strand crosslinking, inter-strand crosslinking and protein crosslinking. This DNA structure change affects the ability of the DNA to be unwound and replicated, resulting in the triggering of the G2-M DNA damage checkpoint and cell cycle arrest. The affected cell will attempt DNA repair and, if unsuccessful, undergo apoptosis [69]. Expected KEGG terms therefore include those relating to apoptosis and DNA damage.

From Figure 2, KEGG pathways highlighted for this group of studies include ten cancer-specific terms and six cancer-related terms. Here italics denote a KEGG term. The *ErbB signalling pathway* has been found to influence in proliferation, migration, differentiation and apoptosis in cancer [70] and overexpression of ERBB1 and ERBB2 have been implicated in head and neck and breast cancers. The *neurotrophin signalling pathway* is known to trigger MAPK and PI3K signalling, affecting differentiation, proliferation and development, and survival, growth, motility and angiogenesis respectively [71]. Altered expression of genes in this pathway has been found to correlate with poorer survival in colon, breast, lung and prostate cancers. Changes in expression of genes relating to *focal adhesion*, which is responsible for attachment of cells to the extracellular matrix, have been implicated in cancer migration, invasion, survival and growth [72]. The *TGF-beta signalling pathway* also regulates many cellular processes, including proliferation, cellular adhesion and motility, coregulation of telomerase function, regulation of apoptosis, angiogenesis, immunosuppression and DNA repair [73]. The *p53 signalling pathway* has many varied links to cancer. This pathway many be triggered by various stress signals and can result in several responses, including cell cycle arrest, apoptosis, the inhibition of angiogenesis and metastasis, and DNA repair [74]. Finally, *nucleotide excision repair* is known to promote cancer development when both up and down regulated. Down-regulation correlates is thought to increases susceptibility to mutation formation and hence the formation of cancer [75], whereas up-regulation has been found to correlate with resistance to platinum as the DNA damage caused by the chemotherapy agent is repaired [76].

The first group of studies considered patients treated with taxanes in addition to platinum. Taxanes act by stabilising tubulin, preventing the microtubule structure formation required for mitosis. This results in cell cycle arrest at the G2/M DNA damage checkpoint and apoptosis. Mechanisms for taxane resistance are, however, not well understood. Two suggested mechanisms include the increased expression of multidrug transporters, and changes in the expression of the *β*-tubulin isoforms [77]. Neither of these mechanisms seem to be enriched in the platinum and taxol group. In addition to the single-agent effects of platinum and taxanes, there is an additional synergistic effect [78]. However, this effect is also not well studied and hence the mechanisms by which this occurs are not clear.

The second group, as seen in Figure 3, was composed of studies applying chemotherapy treatments other than platinum and taxanes. This group is heterogeneous with respect to chemotherapy treatment, and mainly consists of studies reporting treatment as ‘platinum-based’. The other drug explicitly mentioned by studies in this group is cyclophosphamide. This drug is an alkylating agent and acts to form adducts in DNA [79]. This DNA damage triggers the G2/M DNA damage checkpoint, resulting in DNA repair or apoptosis. This suggests that the same DNA repair mechanisms related to platinum treatment are also relevant to cyclophosphamide. For this group, the KEGG pathway analysis shows that the gene set is enriched with 14 pathways related to cancer, in addition to two general cancer-related terms. The *mTOR signalling pathway* is downstream to the PI3K/AKT pathway and regulates growth, proliferation and survival [80]. The *MAPK signalling pathway* controls the cell cycle, and has been found to contribute to the control of proliferation, differentiation, apoptosis, migration and inflammation in cancer [81]. The *chemokine signalling pathway* has been found to regulate growth, survival and migration in addition to its role in inflammation [82]. Angiogenesis and vasculogenesis are known to be regulated by the *VEGF signalling pathway* [83], which is already the target of treatments such as bevacizumab. *Purine metabolism* is required for the production and recycling of adenine and guanine, and hence is required for DNA replication. This process is the target of chemotherapies such as methotrexate. The term *drug metabolism – other enzymes* is partially cancer related; this term refers to five drugs: azathioprine, 6-mercaptopurine, irinotecan, fluorouracil and isoniazid. Of these, two are chemotherapy treatments; irinotecan is a topoisomerase-I inhibitor and fluorouracil acts as a purine analogue. Also featuring in Figure 3 are *apoptosis*, *ErbB signalling pathway*, *focal adhesion*, *neurotrophin signalling pathway*, *B cell receptor signalling pathway* and *Jak-STAT signalling pathway*, all of which are known to be related to cancer.

Overall, the gene sets appear to be enriched for cancer-related resistance mechanisms [84]. However, when combined there is little evidence from this analysis to suggest that the signatures are capturing chemotherapy-specific mechanisms in addition to more general survival pathways. The DNA repair terms may suggest a response to platinum-based treatment, though the down-regulation of these mechanisms is also related to cancer development and resistance in general [85]. It is likely that, due to the varying reliability suggested by the bias analysis and the reported model development techniques, the signal-to-noise ratio of informative genes is low when the gene signatures are combined, preventing the identification of processes of interest.

### Model predictive ability

#### Sensitivity and specificity

The comparison of the success of the various models is difficult, particularly due to the fact that many papers report different metrics as measures of model accuracy. Many of these are also incomplete, not providing enough information to fully describe the model. Ideally, models should be applied to an independent set of samples with known outcomes and performance measures on this data set reported. For classification models an informative set of measures would be positive predictive value, negative predictive value, specificity and sensitivity:

$$\begin{aligned} \text{Sensitivity} &= \frac{n_{\text{true positive}}}{n_{\text{true positive}}+n_{\text{false negative}}}\\ \text{Specificity} &= \frac{n_{\text{true negative}}}{n_{\text{true negative}}+n_{\text{false positive}}}\\ \text{PPV} &= \frac{n_{\text{true positive}}}{n_{\text{true positive}}+n_{\text{false positive}}}\\ \text{NPV} &= \frac{n_{\text{true negative}}}{n_{\text{true negative}}+n_{\text{false negative}}} \end{aligned} $$

where *n*_true positive_ is the number of true positive predictions, *n*_false positive_ is the number of false positive predictions, *n*_true negative_ is the number of true negative predictions and *n*_false negative_ is the number of false negative predictions.

Together these provide information on true positive and negative rates as well as false positive and false negative rates, all of which are important when assessing the performance of a model.

Using the sensitivity and specificity the positive and negative likelihood ratios may be calculated and, using the prevalence of the condition in the test population, the probability of a patient having the condition based on the test results may be found, as in the equations below.

$$\begin{aligned} \text{LR}_{\text{+ve}} &= \frac{\text{sensitivity}}{1-\text{specificity}}\\ \text{LR}_{\text{-ve}}& = \frac{1-\text{sensitivity}}{\text{specificity}}\\ P(\text{Condition}+|\text{Test}+) &= \frac{\frac{P(\text{Condition}+)}{1-P(\text{Condition}+)}\cdot\text{LR}_{\text{+ve}}}{\frac{P(\text{Condition}+)}{1-P(\text{ Condition}+)}\cdot\text{LR}_{\text{+ve}}+1}\\ P(\text{Condition}+|\text{Test}-) &= \frac{\frac{P(\text{Condition}-)}{1-P(\text{Condition}-)}\cdot\text{LR}_{\text{-ve}}}{\frac{P(\text{Condition}-)}{1-P(\text{Condition}-)}\cdot\text{LR}_{\text{-ve}}+1} \end{aligned} $$

These post-test probabilities are much easier to interpret and incorporate the prevalence of the condition. It should be noted that in order for the test to be applied in a clinical situation the pre-test probabilities used, *P*(Condition+) and *P*(Condition−), should be correct for the population of patients to whom the test will be applied. Here the sample prevalence from each study was used for convenience. However, it would be informative to recalculate *P*(Condition+|Test+) and *P*(Condition+|Test−) for the general population of ovarian cancer patients, as this would provide a better comparison between models.

Table 12 details the post-test probabilities of patients having a condition based on a positive or negative test result from the models developed by studies in this review. The papers appearing here are those that supplied sensitivity and specificity and the numbers of patients with and with without the condition, or alternative information allowing these to be calculated such as numbers of true and false positives and negatives.

**Table 12:** **Prediction metrics for studies reporting sensitivity and specificity**

Study	Prediction	Sensitivity	Specificity	LR_+ve_^*†*^	LR_-ve_^*†*^	*P*(C+)^*†*^	*P*(C−)^*†*^	*P*(C+|T+)^*†*^	*P*(C+|T−)^*†*^
Li *et al.* [3]	Chemoresistance	0.96^*^	0.23^*^	1.24	0.18	$\frac {22}{44}$	$\frac {22}{44}$	0.55	0.15
Obermayr *et al.* [27]	RFS	0.22^*^	0.85^*^	1.47	0.92	$\frac {46}{216}$	$\frac {170}{216}$	0.28	0.77
Ferriss *et al.* [33]	Chemoresponse	0.94^*^	0.29^*^	1.33	0.20	$\frac {85}{119}$	$\frac {34}{119}$	0.77	0.07
Sabatier *et al.* [37]	Prognosis	0.62^*^	0.62^*^	1.64	0.62	$\frac {194}{366}$	$\frac {172}{366}$	0.65	0.35
Yoshihara *et al.* [43]	PFS	0.64^*^	0.69^*^	2.06	0.52	$\frac {45}{87}$	$\frac {39}{87}$	0.69	0.30
Williams *et al.* [44]	Prognosis	0.77^*^	0.56^*^	1.75	0.41	$\frac {97}{143}$	$\frac {46}{143}$	0.79	0.16
Gevaert *et al.* [49]	Chemoresistance	0.67^*^	0.40^*^	1.12	0.82	$\frac {15}{45}$	$\frac {30}{45}$	0.36	0.62
Helleman *et al.* [53]	Chemoresistance	0.89^*^	0.56^*^	2.02	0.20	$\frac {9}{72}$	$\frac {63}{72}$	0.22	0.58
De Smet *et al.* [52]	Chemoresistance	0.71^*†*^	0.83^*†*^	4.29	0.34	$\frac {6}{13}$	$\frac {7}{13}$	0.79	0.29
Raspollini *et al.* [56]	Prognosis	0.79^*†*^	0.46^*†*^	1.45	0.47	$\frac {28}{52}$	$\frac {24}{52}$	0.63	0.29
Hartmann *et al.* [57]	Prognosis	0.86^*^	0.86^*^	6.14	0.16	$\frac {21}{28}$	$\frac {7}{28}$	0.95	0.05
Selvanayagam *et al.* [59]	Chemoresistance	1.00^*†*^	1.00^*†*^	*∞*	0.00	$\frac {4}{8}$	$\frac {4}{8}$	1.00	0.00
Kamazawa *et al.* [61]	Chemoresponse	1.00^*^	0.83^*†*^	6.00	0.00	$\frac {21}{27}$	$\frac {5}{27}$	0.95	0.00

^*^Value stated in reference.

^†^Value calculated.

C: condition presence.

T: test result.

RFS: Relapse Free Survival.

PFS: Progression Free Survival.

From the table it may be seen that there is a great variety between the success of the models. For example, Kamazawa *et al.* [61] and Hartmann *et al.* [57] both achieved *P*(Condition+|Test+)=0.95 on their respective samples of the population. This means that if a patient tests positive, there is a 95% probability that they are positive for the condition in question, which in these cases are ‘responding to chemotherapy’ and ‘poor prognosis’ respectively. In contrast, Obermayr *et al.* [27], Helleman *et al.* [53] and Gevaert *et al.* [49] only achieved *P*(Condition+|Test+) of between 0.20 and 0.40. These results suggest that the tests are not able to predict the outcome of a patient any better than a random choice, and in the case of tests in the region of 0.20 it is likely that most patients are simply assigned to the same class.

The ability of tests to not commit type II errors and give false negatives is also important. Ferriss *et al.* [33] and Hartmann *et al.* [57] both achieved well in this regard, with *P*(Condition+|Test−)=0.07 and *P*(Condition+|Test−)=0.05 respectively. Several studies, by contrast, had very poor probabilities of false negatives; Obermayr *et al.* [27], Helleman *et al.* [53] and Gevaert *et al.* [49] all have *P*(Condition+|Test−)>0.5, which suggests that these models give a false negative more often than a random assignment.

Kamazawa *et al.* [61] and Selvanayagam *et al.* [59] both achieved extremely impressive prediction abilities, as may be seen by the very large *P*(Condition+|Test+) and very small *P*(Condition+|Test−) values. However, these studies exemplify why care must be taken in assessing the predictive ability of models. Both studies calculated sensitivity and specificity based on only training set results and hence there is no way to judge the generalisability of the models. There is a tendency for models to perform better on the training set than any following independent data set to which it is subsequently applied. Secondly, the training set used by Selvanayagam *et al.* [59] is extremely small at eight patients and has a 50 : 50 ratio of chemoresistant to chemosensitive patients. This sample is not representative of the population and hence the values of *P*(Condition+|Test+) and *P*(Condition+|Test−) will be skewed by unrepresentative *P*(Condition+) and *P*(Condition−).

Overall, the most successful model of this group is that by Hartmann *et al.* [57] as it makes predictions with good reliability and has been validated on an independent data set. The least successful models were Obermayr *et al.* [27], Helleman *et al.* [53] and Gevaert *et al.* [49]. These studies suffered from low ability to identify true positives and high probability of false positives, resulting in poor predictive ability.

#### Hazard ratios

It is common for studies of survival to quote hazard ratios comparing the results of clusters identified by classification models or relative-risk models such as Cox proportional hazards regression. These ratios represent the ratio of the probability of an event occurring to a patient in each of the two groups. The event is often death, but could also be recurrence for example. The studies listed in Table 13 supplied hazard ratios as measures of predictive ability. The hazard ratios vary from 0.23 to 4.6 with the majority around 2 to 3. A hazard ratio that is not equal to 1 suggests that the variable has predictive ability, and a ratio of 4, for example, suggests that a member of the high-risk group is 4 times as likely to die within the study period than a member of the low-risk group. The study with the highest hazard ratio is Spentzos *et al.* [58], with HR=4.6. This is closely followed by Raspollini [56] with HR=0.23 and Skirnisdottir and Seidal [35] with HR=4.12. The confidence intervals on the hazard ratios of all the studies are large and, with the exception of Spentzos *et al.* [58], at the lowest edge the hazard ratio is very close to 1. This suggests that, although all these hazard ratios were found to be significant, some were close to not reaching the arbitrary 5*%* level. Most notable are Roque *et al.* [24], Schlumbrecht and Seidal[40], and Denkert *et al.* [45]. These models would need further investigation to determine their predictive ability. Of the papers in this group, Spentzos *et al.* [58] appears to have the best predictive ability when classifying patients into two clusters with significantly different survival times.

**Table 13:** **Prediction metrics for studies reporting hazard ratios**

Study	Prediction	Classes	HR	95% CI	Median survival	P value
Jeong *et al.* [22]	OS	YA subgroup vs. YI subgroup	0.5	0.31−0.82		0.005
Roque *et al.* [24]	OS	High vs. low TUBB3 staining	3.66	1.11−12.05	707 days vs. not reached	0.03
Kang *et al.* [31]	OS	High vs. low score	0.33	0.13−0.86	1.8 years vs. 2.9 years	<0.001
Skirnisdottir and Seidal [35]	Recurrence	p53 -ve vs. +ve	4.12	1.41−12.03		0.009
Schlumbrecht *et al.* [40]	RFS	EIG121 high vs. low	1.13	1.02−1.26		0.021
Yoshihara *et al.* [43]	PFS	High vs. low score	1.64	1.27−2.13		0.0001
Denkert *et al.* [45]	OS	Low vs. high score	1.7	1.1−2.6		0.021
Crijns *et. al* [47]	OS		1.94	1.19−3.16		0.008
Netinatsunthorn *et al.* [51]	RFS	Yes vs. no WT1 staining	3.36	1.60−7.03		0.0017
Spentzos *et al.* [54]	OS	Resistant vs. sensitive	3.9	1.3−11.4	41 months vs. not reached	<0.001^*†*^
Raspollini *et al.* [56]	OS	No vs. yes COX-2 staining	0.23	0.06−0.77		0.017
Spentzos *et al.* [58]	OS	High vs. low score	4.6	2.0−10.7	30 months vs. not reached	0.0001

^†^Calculated value.

HR: Hazard Ratio.

OS: Overall Survival.

RFS: Relapse Free Survival.

PFS: Progression Free Survival.

CI: Confidence Interval.

#### Linear regression

Two papers reported the success of model assessed using linear regression: Glaysher *et al.* [41] and Kang *et al.* [31]. These studies plotted the predicted values or model score against the measured values and applied linear regression to obtain a line of best fit. The *R*^2^ or $R^{2}_{\text {adj}}$ of this line is then calculated to assess the discrimination of the model. Glaysher *et al.* [41] achieved *R*^2^=0.901 ($R^{2}_{\text {adj}}=0.836$) for a model predicting resistance to cisplatin via cross-validation and Kang *et al.* [31] achieved *R*^2^=0.84 for a model predicting recurrence-free survival in the data set on which it was derived. These values suggest a good level of predictive ability, both in terms of calibration and discrimination, with the model by Glaysher *et al.* [41] achieving the better predictions.

#### Cox proportional hazards models

When studies identified by this review applied the Cox proportional hazards model to predict patient outcome, it was common for the main analysis of the model to be assessing whether the gene signature was found to be significant and whether the signature was an independent predictor. However, the application of this model to an independent data set was much less common. As may be seen from Table 6, the success of many models was judged using the significance of covariates including the gene signature in the model. It is likely that this model was not applied to external data sets due to subtleties in what the model predicts when compared to methods such as linear regression. Whereas in linear regression the survival times are predicted directly, Cox proportional hazards regression predicts hazard ratios. Royston and Altman [86] developed techniques for the external validation of Cox proportional hazards models by application to an independent data set. These rely on having at least the weights of the variables included in the linear predictor, and ideally the baseline survival function. The first allows the assessment of the discriminatory power of a model, whereas the second is also required to allow the calibration of the model to be assessed. Royston and Altman [86] are of the opinion that the inclusion of a log-rank test p-value is not informative due to the irrelevance of the null hypothesis being tested, and hence this should not be considered when judging model performance. An alternative to the log-rank test to compare survival between groups would be time-dependent ROC curves [87].

#### Failure to predict

Of the studies identified by this review, some models failed to achieve significant predictive ability. These include Lisowska *et al.* [23], Vogt *et al.* [62] and Brun *et al.* [34]. Of these papers, Vogt *et al.* [62] and Brun *et al.* [34] both considered small numbers of genes when constructing their models. It is possible then that these models failed because no informative genes were considered. Conversely, Lisowska [23] applied their modelling technique to over 47000 genes using 127 patients. It is therefore a possibility that genes were selected by their model purely by chance rather than due to true explanatory ability. This model was tested using an independent data. When the model was applied to this data set it performed poorly, suggesting that the genes chosen did not generalise to the second cohort of patients. Neither Vogt *et al.* [62] nor Brun *et al.* [34] reported measuring the precision or accuracy of the gene expression measurements. Lisowska *et al.* [23] used RT-PCR to measure the expression of 18 genes from the microarray, but the RT-PCR measurements were carried out on a separate set of samples and hence are not useful when considering accuracy. It is therefore unknown whether the gene expression measurement techniques applied by these studies were sufficiently accurate.

## Discussion

The papers identified as part of this review tackled the important issue of chemoresistance and survival prediction in ovarian cancer via gene or protein expression. The concept of identifying gene signatures is popular, but requires careful handling to extract the information required for this to be successful. It was observed that of the many different tissue preservation techniques applied, the most common were fresh-frozen and formalin fixed, paraffin embedded tissue. It is our opinion that, due to the high quality expression measurements that may now be achieved with FFPE tissue, this is the most appropriate choice for research intended to translate into a clinical setting.

It was found that the majority of the studies included in this review were heterogeneous with respect to the histological type of the patient cohort. This suggests that, due to the differing response of different types of ovarian cancer to chemotherapy, the gene signatures may be identifying different pathways and mechanisms. However, it should also be noted that although 27 of the 42 studies were heterogeneous, 12 of these consisted of greater than 80% serous samples. Therefore, for these studies the inclusion of multiple histological types is likely to have less effect on the gene signature and mechanisms highlighted could be expected to occur in serous ovarian cancer. It would be advisable for future studies to include histological type and grade as model features.

The majority of studies identified by this review attempt to classify patients into groups with different characteristics, for example ‘poor prognosis’ and ‘good prognosis’ or ‘chemosensitive’ and ‘chemoresistant’. However, variables such as response to chemotherapy and prognosis are rarely so well separated into classes; they are by nature continuous variables. Altman and Royston [88] are clear that dichotomising continuous variables into categories (such as high-risk vs. low-risk) should be avoided, as it results in loss of information and may lead to underestimation of variation and the masking of non-linearity. Arbitrary choices of cutoff values may further obscure the situation, when the original continuous variable could serve the same purpose in many models. In terms of a clinical test it therefore may be more appropriate to apply alternative techniques, such as various types of regression, to obtain a real valued prediction of patient outcome.

It was noted that the metrics reported as measures of predictive ability vary between studies. These vary in the amount of information conveyed and hence care should be taken to use metrics that fully describe the model. Sensitivity and specificity are commonly reported for classification techniques and, together with the numbers of patients in each class in the data set, allows the probabilities of a patient having the condition of interest given that they have tested positive or negative. It is the ultimate aim of most classification studies to obtain these probabilities, as it allows the predictive ability of the test to be assessed and the applicability of the test to be evaluated. Of the studies reporting sensitivity, specificity and related information, the best predictive ability was achieved by Hartmann *et al.* [57] and the worst by Helleman *et al.* [53]. It is important to note that from the sensitivity and specificity the model by Helleman *et al.* [53] does not appear to be any worse than some of the others, but these probabilities incorporate the prevalence of the condition of interest in the test population. It would therefore be highly informative to recalculate these probabilities using the prevalence of the condition in the population of ovarian cancer patients. Since some of the test populations were not representative of the overall population (having so called ‘spectrum bias’), this would give a much more reliable indication of the predictive ability of the models in a clinical setting.

One of the main aims of the studies identified was to obtain a ‘gene signature’, the expression of which can explain and predict the response in the patient. To this end, the majority of the papers (32 of 42) provided full or partial list of the genes selected by the modelling process. An analysis of these gene signatures resulted in the conclusion that the signatures were very dissimilar, with the most commonly selected gene appearing in only four papers. 93.53% of genes were selected by only one paper. This seems to indicate that the gene signatures identified were not based on underlying cellular processes, or at least that the processes being highlighted were not the same across the papers. It should be noted that many of the studies used cohorts of patients who were heterogeneous in terms of chemotherapy treatment and, due to the development of resistance to chemotherapy via gene expression changes, this may affect the genes found to be explanatory. It may be that several gene signatures from sub-populations of patients treated with different drugs are combining and hence reducing the predictive ability of the models.

In order to assess the biological relevance of the genes selected for the gene signatures, gene set enrichment analysis was carried out. This technique is used to highlight processes and pathways that are over-represented in the gene signature compared to the set of all genes. For the purposes of this review, two groups of studies were considered: those where the patients were treated with platinum and taxane, and those where the patients were treated with other platinum based treatments. These groups were selected due to the low numbers of studies using a single treatment option. For example, there were no studies considering platinum, taxane or cyclophosphamide as single agents. Following the analysis, 30 KEGG terms were returned for each group. Of these, each list comprised of approximately half cancer related terms. Of these the majority were processes often up- or down-regulated in cancer cells, such as proliferation, apoptosis, and motility and metastasis [89]. It is unclear whether the change in regulation of these processes is further altered in response to specific chemotherapy treatments. However, one process worthy of additional consideration is DNA repair. DNA repair is known to be an important mechanism in cancer both though cancer development when down-regulated or mutated [75] and resistance to DNA damaging chemotherapy when up-regulated [76]. Therefore, the strong presence of DNA repair terms may suggest the presence of platinum resistance pathways in the gene signatures. It is the authors’ opinion that, although the combined gene signatures appear not to include predictive chemotherapy-specific information, they may be capable of providing prognostic information. It is also thought that some studies, such as Glaysher *et al.*, may include genes relevant to additional chemotherapy-specific processes which are ‘drowned out’ when combined with other signatures.

## Conclusion

It is clear that the prediction of response to chemotherapy in ovarian cancer is an ongoing research problem that has been attracting attention for many years. However, although many studies have been published, a clinical tool is still not available. It is our belief that, although not yet accomplished, progress within the field suggests that the development of a predictive model is possible. There is great variability between the approaches and success of existing studies in the literature, and there have been very high levels of variation in the genes identified as explanatory. It is the authors’ opinion that, if more care is taken when selecting the patients for inclusion to control for treatment history, these gene signatures may be simplified and models able to predict response to treatment may be developed.

## Additional files

Additional file 1
**PubMed search terms.**

Additional file 2
**PRISMA Checklist.**

Additional file 3
**Bias assessment, including QUADAS-2 and CEBM levels of evidence.**
