# Supplementary material for: Prediction of resistance to chemotherapy in ovarian cancer: a systematic review
Source: BMC Cancer. 2015 Mar 11;15:117. doi: 10.1186/s12885-015-1101-8 (PMC4371880; doi:10.1186/s12885-015-1101-8)
Supplement: Additional file 3 — Bias assessment, including QUADAS-2 and CEBM levels of evidence. [file 12885_2015_1101_MOESM3_ESM.pdf]

QUADAS-2 [18] **results and CEBM 2011 Levels of Evidence** [19]

Study	RISK OF BIAS				APPLICABILITY CONCERNS			Level of Evidence
	PATIENT SELECTION	INDEX TEST	REFERENCE STANDARD	FLOW AND TIMING	PATIENT SELECTION	INDEX TEST	REFERENCE STANDARD	
Jeong *et. al.* [27]	☺	☺	☺	☺	☺	☺	☺	2
Lisowska *et. al.* [44]	☺	☺	☺	☺	☺	☺	☺	2
Roque *et. al.* [39]	☺	?	☺	☺	☺	☺	☺	2
Li *et. al.* [3]	☺	☺	☺	☺	☺	☺	☺	2
Schwede *et. al.* [47]	☺	☺	☺	☺	☺	☺	☺	2
Verhaak *et. al.* [48]	☺	☺	☺	☺	☺	☺	☺	2
Obermayr *et. al.* [32]	☺	☺	☺	☺	☺	☹	☺	2
Han *et. al.* [49]	☺	☺	☺	☺	☺	☺	☺	2
Hsu *et. al.* [50]	☺	☺	☺	☺	☺	☺	☺	2
Lui *et. al.* [51]	☺	☺	☺	☺	☺	☺	☺	2
Kang *et. al.* [42]	☺	☺	☺	☺	☺	☺	☺	2
Gillet *et. al.* [52]	☺	☺	☺	☺	☺	☺	☺	2
Ferriss *et. al.* [28]	☺	☺	☺	☺	☺	☺	☺	2
Brun *et. al.* [45]	☺	☺	☺	☺	☺	☺	☺	2
Skirnisdottir and Seidal [38]	☺	?	☺	☺	☺	☺	☺	2
Brenne *et. al.* [53]	☺	☺	☺	☺	☺	☺	☺	2
Sabatier *et. al.* [54]	☺	☺	☺	☺	☺	☺	☺	2
Gillet *et. al.* [24]	☺	☺	☺	☺	☺	☺	☺	2
Chao *et. al.* [55]	☹	☺	☹	☺	☺	☺	☹	3
Schlumbrecht *et. al.* [40]	☺	☺	☺	☺	☺	☺	☺	2
Glaysher *et. al.* [20]	☺	☺	☺	☺	☺	☺	☺	2
Yan *et. al.* [56]	☺	☺	☺	☺	☺	☺	☺	2
Yoshihara *et. al.* [22]	☺	☺	☺	☺	☺	☺	☺	2
Williams *et. al.* [29]	☺	☺	☺	☺	☺	☺	☺	2
Denkert *et. al.* [41]	☺	☺	☺	☺	☺	☺	☺	2
Matsumara *et. al.* [30]	☺	☺	☺	☺	☺	☺	☺	2
Crijns *et. al.* [23]	☺	☺	☺	☺	☺	☺	☺	2
Mendiola *et. al.* [57]	?	?	☺	☺	☺	☺	☺	2
Gevaert *et. al.* [34]	?	☺	☺	☺	☺	☺	☺	2
Bachvarov *et. al.* [58]	☹	☺	☺	☺	☺	☺	☺	3
Netinatsunthorn *et. al.* [59]	☺	☺	☺	☺	☺	☺	☺	2
De Smet *et. al.* [60]	☹	☺	☺	☺	☺	☺	☺	3
Helleman *et. al.* [33]	☺	☺	☺	☺	☺	☺	☺	2
Spentzos *et. al.* [61]	☺	☺	☺	☹	☺	☺	☺	2
Jazaeri *et. al.* [62]	☹	☺	☺	☺	☺	☺	☺	3
Raspollini *et. al.* [37]	?	☺	☺	☺	☺	☺	☺	3
Hartmann *et. al.* [21]	☺	☺	☺	☺	☺	☺	☺	2
Spentzos *et. al.* [36]	☺	☺	☺	☺	☺	☺	☺	2
Selvanayagam *et. al.* [35]	?	☺	☺	☺	☺	☺	☺	3
Iba *et. al.* [63]	☺	☺	☺	☺	☺	☺	☺	2
Kamazawa *et. al.* [31]	☺	☹	☺	☺	☺	☺	☺	2
Vogt *et. al.* [26]	☺	☺	☺	☺	☺	☺	☺	2

☺ Low Risk   ☹ High Risk   ? Unclear Risk

Table modified from http://www.bris.ac.uk/quadas/resources/
